# Phytochemistry and Pharmacology of *Eleutherococcus sessiliflorus* (Rupr. & Maxim.) S.Y.Hu: A Review

**DOI:** 10.3390/molecules28186564

**Published:** 2023-09-11

**Authors:** Hui Sun, Jiaxin Feng, Yue Sun, Shuang Sun, Li Li, Junyi Zhu, Hao Zang

**Affiliations:** 1 Green Medicinal Chemistry Laboratory, School of Pharmacy and Medicine, Tonghua Normal University, Tonghua 134002, China; sunhui9405@163.com (H.S.); 13630304082@163.com (J.F.); 15981309048@163.com (Y.S.); ss11721@163.com (S.S.); swx0527@thnu.edu.cn (J.Z.); 2 College of Pharmacy, Yanbian University, Yanji 133000, China; 3 Key Laboratory of Evaluation and Application of Changbai Mountain Biological Gerplasm Resources of Jilin Province, Tonghua 134002, China

**Keywords:** *Eleutherococcus sessiliflorus* (Rupr. & Maxim.) S.Y.Hu, herbal metabolites, pharmacological effects, triterpenoids

## Abstract

*Eleutherococcus sessiliflorus* (Rupr. & Maxim.) S.Y.Hu (*E. sessiliflorus*), a member of the Araliaceae family, is a valuable plant widely used for medicinal and dietary purposes. The tender shoots of *E. sessiliflorus* are commonly consumed as a staple wild vegetable. The fruits of *E. sessiliflorus*, known for their rich flavor, play a crucial role in the production of beverages and fruit wines. The root barks of *E. sessiliflorus* are renowned for their therapeutic effects, including dispelling wind and dampness, strengthening tendons and bones, promoting blood circulation, and removing stasis. To compile a comprehensive collection of information on *E. sessiliflorus*, extensive searches were conducted in databases such as Web of Science, PubMed, ProQuest, and CNKI. This review aims to provide a detailed exposition of *E. sessiliflorus* from various perspectives, including phytochemistry and pharmacological effects, to lay a solid foundation for further investigations into its potential uses. Moreover, this review aims to introduce innovative ideas for the rational utilization of *E. sessiliflorus* resources and the efficient development of related products. To date, a total of 314 compounds have been isolated and identified from *E. sessiliflorus*, encompassing terpenoids, phenylpropanoids, flavonoids, volatile oils, organic acids and their esters, nitrogenous compounds, quinones, phenolics, and carbohydrates. Among these, triterpenoids and phenylpropanoids are the primary bioactive components, with *E. sessiliflorus* containing unique 3,4-seco-lupane triterpenoids. These compounds have demonstrated promising properties such as anti-oxidative stress, anti-aging, antiplatelet aggregation, and antitumor effects. Additionally, they show potential in improving glucose metabolism, cardiovascular systems, and immune systems. Despite some existing basic research on *E. sessiliflorus*, further investigations are required to enhance our understanding of its mechanisms of action, quality assessment, and formulation studies. A more comprehensive investigation into *E. sessiliflorus* is warranted to delve deeper into its mechanisms of action and potentially expand its pharmaceutical resources, thus facilitating its development and utilization.

## 1. Introduction

**Figure 1:**
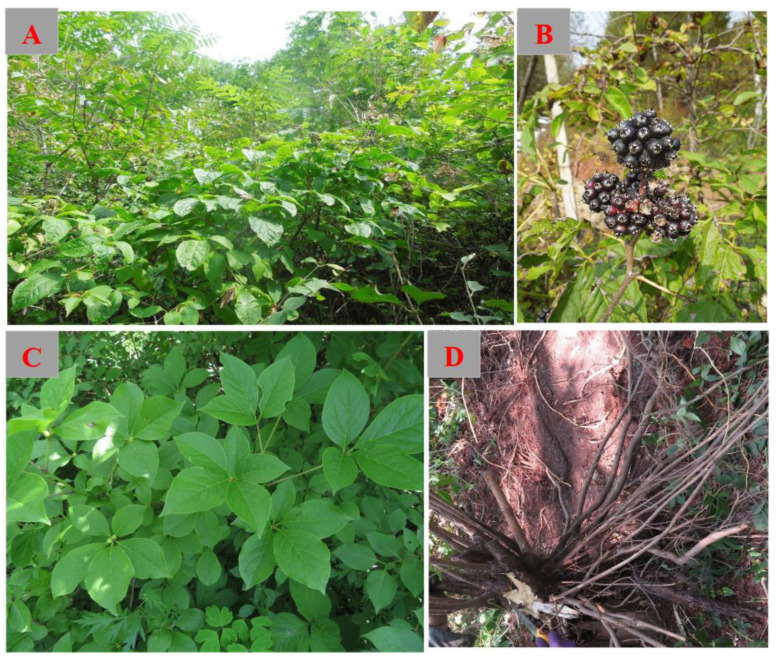
Whole plant (**A**), fruits (**B**), leaves (**C**), and roots (**D**) of *Eleutherococcus sessiliflorus.*

*Eleutherococcus sessiliflorus* (Rupr. & Maxim.) S.Y.Hu (*E. sessiliflorus*), also known as *Acanthopanax sessiliflorus* or “Ciguaibang” in Chinese, is a plant belonging to the Araliaceae family, specifically within the *Eleutherococcus* genus. It has a wide distribution across various regions of China, including Jilin, Heilongjiang, Liaoning, and Hebei. It is also dispersed in neighboring countries like North and South Korea, Japan, and the Russian Far East [1,2]. More recently, Poland has also shown interest in *E. sessiliflorus* and has started introducing and cultivating it [3]. *E. sessiliflorus* thrives in forests and shrublands at altitudes ranging from 200 to 1000 m (Figure 1). *E. sessiliflorus* plants resemble another medicinal plant called *Eleutherococcus senticosus* (*E. senticosus*) (also known as Siberian ginseng), often leading to confusion between the two. However, a notable distinction lies in the absence of thorns on *E. sessiliflorus* branches and the presence of short, nearly spherical fruit stalks.

The pharmacological effects of *E. sessiliflorus* have been recognized for centuries, with early records in ancient Chinese medicinal texts such as “Shennong’s Classic Materia Medica” (Shennong Bencao Jing) and the “Compendium of Materia Medica” (Bencao Gangmu) [4]. *E. sessiliflorus* has been revered as a high-quality herb, often described in phrases like “Prefer a handful of *Eleutherococcus* over a cartload of gold and jade.” Notably, the root barks of *E. sessiliflorus* are documented in the “Chinese Materia Medica” (Zhonghua Bencao) and “Dictionary of Traditional Chinese Medicine” (Zhongyao Dacidian) as a source of Wujia Pi. *E. sessiliflorus* is known for its properties in dispelling wind-dampness, strengthening tendons and bones, and promoting blood circulation while removing stasis [5]. It has been traditionally used to address conditions such as anemofrigid-damp arthralgia, sudden muscle contractions and spasms, lower back pain, impotence, weak lower limbs, delayed childhood mobility, edema, dermatophytosis, ulcers, abscesses, swellings, and traumatic injuries [5]. Furthermore, the tender stems of *E. sessiliflorus* hold culinary value and are consumed as a wild vegetable. With their crisp and tender texture, delightful taste, unique flavor, and abundant nutrition, they have been a part of the northeastern mountainous region’s diet for centuries.

Extensive research has investigated and isolated over 300 compounds from *E. sessiliflorus*, spanning a wide range of compound categories. These include monoterpenoids, sesquiterpenoids, and triterpenoids, as well as simple phenylpropanoids, lignans, coumarins, flavonoids, volatile oils, organic acids and their esters, nitrogenous compounds, quinones, phenolics, and carbohydrates. Notably, triterpenoids and lignans have gained significant attention in chemical composition research due to their remarkable potential for further research and development [6,7,8,9]. Since its approval as a new food resource by the Ministry of Health of the People’s Republic of China in 2008, *E. sessiliflorus* has become the foundation for a series of health products, such as tea, concentrated solutions, and wines, continuously entering the market. Modern pharmacological research has uncovered the remarkable properties of *E. sessiliflorus*, including its anti-inflammatory, antioxidant, antiplatelet aggregation, vasodilatory, cardioprotective, anti-aging, and anticancer effects [7,8,10,11,12]. Additionally, *E. sessiliflorus* can potentially improve glucose metabolism, cardiovascular function, and immune response.

Despite existing research that has summarized the phytochemistry and pharmacological effects of *E. sessiliflorus*, there are notable deficiencies in comprehensive coverage. These deficiencies include incomplete classification of components, partial listing of constituents, and a lack of chemical structure information for these components. Additionally, the mechanisms underlying the pharmacological effects are often inadequately detailed and clarified. Although there is a report that provides a good overview of the traditional uses, secondary metabolites, and pharmacology of *Eleutherococcus* species [13], the limited space may have resulted in the report only listing a few triterpenoids, certain lignins, and a limited number of flavonoids in *E. sessiliflorus*, totaling approximately 50 components. In contrast, our review reports a total of 314 components and provides structural information for each of these components.

Furthermore, our review focuses on a different classification of pharmacological research compared to the aforementioned report. Lastly, our review incorporates research findings on *E. sessiliflorus* from the past two years, offering a more up-to-date and comprehensive perspective. Therefore, this review aims to fill this gap by providing a comprehensive review of the phytochemistry and pharmacological effects of *E. sessiliflorus*. The goal of this endeavor is to serve as a valuable reference for future investigations into *E. sessiliflorus*, while also providing new insights for the rational utilization of *E. sessiliflorus* resources and the efficient development of related products.

## 2. Phytochemistry

There is a growing interest in utilizing *E. sessiliflorus* as a novel resource in the food industry, given the numerous bioactive compounds that have been successfully isolated and identified from its roots, stems, leaves, and fruits. The fact that it contains a large amount of triterpenoids, phenylpropanoids, and flavonoids highlights the rich potential of *E. sessiliflorus* as a valuable source of bioactive compounds for further exploration in functional food and nutraceutical applications.

### 2.1. Terpenoids

**Figure 2:**
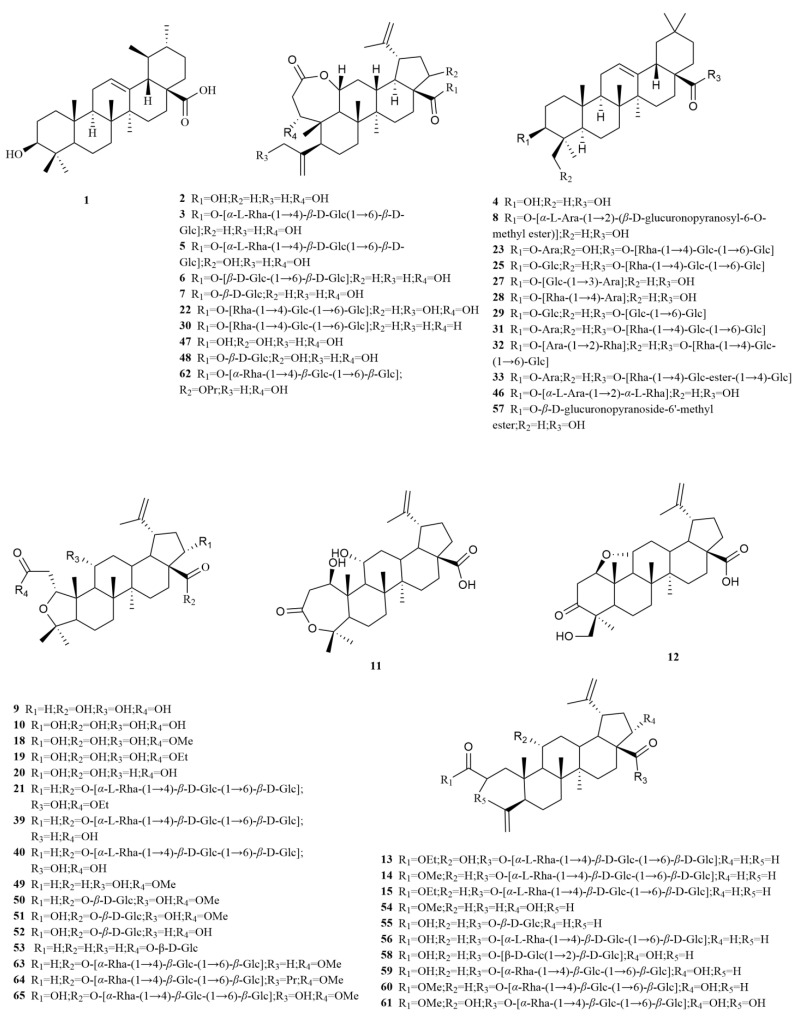

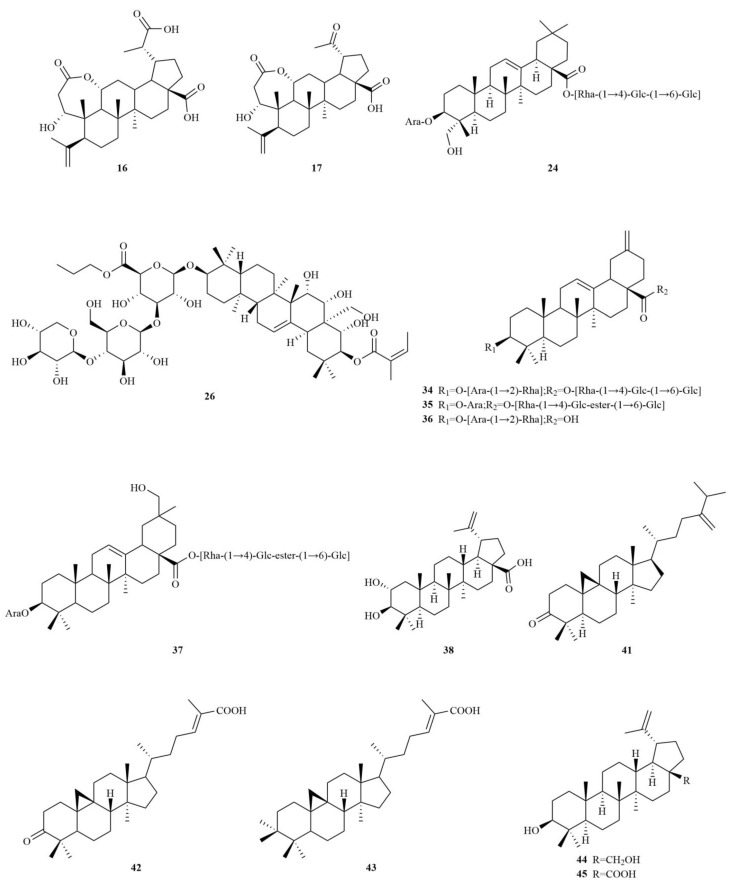

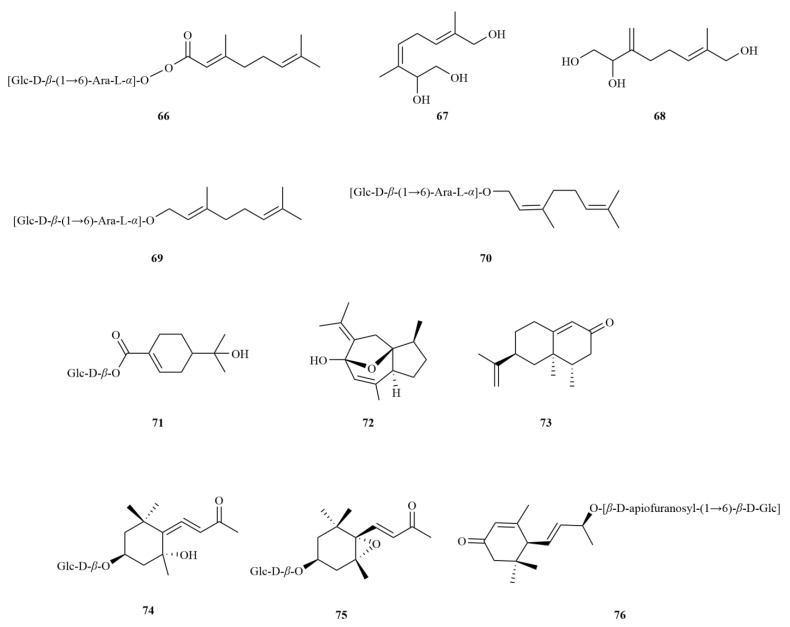
Chemical structures of terpenoids isolated from *Eleutherococcus sessiliflorus.* Chemical structures were drawn using Chemdraw Professional 15.0 software.

*E. sessiliflorus* primarily consists of triterpenoids and their saponins, with smaller amounts of monoterpenoids and sesquiterpenoids. The triterpenoids are predominantly isolated from the fruits and leaves, leading to the identification of seventy-six triterpenoids and their saponins (Table 1, Figure 2). These compounds mainly belong to the oleanane-type, lupane-type, and 3,4-seco-lupane triterpenoid categories. Notably, the 3,4-seco-lupane triterpenoids are unique to the Eleutherococcus genus, with the first 3,4-seco-lupane triterpenoid saponin being isolated in 1985 [14]. Later, this type of compound has been discovered in various Eleutherococcus species. Within *E. sessiliflorus*, thirty-six 3,4-seco-lupane triterpenoids have been isolated and identified, including compounds like chiisanogenin (**2**), chiisanoside (**3**), elesesterpene C (**13**), elesesterpene D (**14**), elesesterpene H (**18**), elesesterpene I (**19**) [15,16]. Chiisanogenin and chiisanoside produced significant anti-inflammatory effects at doses of 10 and 30 mg/kg, with the effect of chiisanogenin being superior to chiisanoside [17].

Among these triterpenoids, elesesterpenes A-K, isolated from the leaves, have garnered significant attention due to their remarkable anti-inflammatory activity. Moreover, these compounds exhibit significant anti-proliferative effects on various human cancer cell lines, including hepatocellular carcinomas (HepG2), lung adenocarcinoma (A549), and glioblastoma (LN229) [16]. On the other hand, acanthosessiliosides A-O, isolated from the fruits, have demonstrated inhibitory effects on lipopolysaccharide-induced RAW264.7 cells. Additionally, some of these compounds have exhibited inhibitory activities against six different human cancer cell lines, including colon adenocarcinoma, breast adenocarcinoma, ovarian adenocarcinoma, cervix adenocarcinoma, hepatoma, and melanoma [7,9]. The lupane-type compounds in *E. sessiliflorus* include betulin (**44**) and betulinic acid (**45**). Additionally, the oleanane-type compounds comprise oleanolic acid (**4**), 3-O-[(α-L-arabinopyranosyl)-(1→2)]-[β-D-glucuronopyranosyl-6-O-methyl ester]-olean-12-ene-28-olic acid (**8**), hederacoside D (**23**) [18,19,20] (Table 1, Figure 2), and numerous others. In cell-based studies, betulin, betulinic acid, and oleanolic acid potentiated *β*-cell function and mass and enhanced hepatic insulin sensitivity to regulate blood sugar [21]. 

Furthermore, the first identified ursane-type triterpenoid in *E. sessiliflorus* is ursolic acid (**1**), predominantly found in fruits [22]. Ursolic acid (100 mg/kg) reduces myocardial damage by inhibiting oxidative stress [23]. Triterpenoids have been extensively studied and have demonstrated remarkable pharmacological effects, including anti-inflammatory, antioxidant, anti-platelet aggregation, vasodilatory, and cardioprotective properties. Furthermore, pharmacokinetic studies have contributed to elucidating the mechanisms of action of *E. sessiliflorus* and have guided its clinical applications [24]. In comparison, research on monoterpenoids within *E. sessiliflorus* is relatively limited. Six monoterpenoids have been isolated from the ethanol extract of fruits, comprising five acyclic monoterpenoids and one cyclic monoterpenoid [25]. Additionally, two sesquiterpenoids, curcumenol (**72**), and nootkatone (**73**), have been identified from the leaves [20]. Curcumenol (2.5–20 μM) possesses potential anti-inflammatory activities by diminishing pro-inflammatory mediators and cytokines and suppressing the expression of regulatory proteins [26]. Nootkatone (10 mg/kg) could inhibit acute and chronic inflammatory responses in mice [27].

**Table 1:** Terpenoids isolated from *Eleutherococcus sessiliflorus*.

NO.	Name	Formula	The exact Theoretical Molecular Weight	Source	Characterization Method	Refs.
**1**	Ursolic acid	C_30_H_48_O_3_	456.3603	fruits, leaves	IR, ^13^C-NMR, ^1^H-NMR	[22,28]
**2**	Chiisanogenin	C_30_H_44_O_5_	484.3189	fruits, leaves	IR, ^13^C-NMR, ^1^H-NMR	[15]
**3**	Chiisanoside	C_48_H_74_O_19_	954.4824	fruits, leaves	IR, ^13^C-NMR, ^1^H-NMR	[15]
**4**	Oleanolic acid	C_30_H_48_O_3_	456.3603	fruits, leaves, roots	HPLC, HPLC-MS	[18,28,29]
**5**	22*α*-Hydroxychiisanoside	C_48_H_74_O_20_	970.4773	fruits	HPLC-MS, ^13^C-NMR, ^1^H-NMR	[30]
**6**	Divaroside	C_43_H_68_O_15_	824.4558	leaves	HPLC	[10]
**7**	Sessiloside-A1	C_36_H_54_O_10_	646.3717	leaves	HPLC, UV, IR, HR-MS	[10,31]
**8**	3-O-[(*α*-L-Arabinopyranosyl)-(1→2)]-[*β*-D-glucuronopyranosyl-6-O-methyl ester]-olean-12-ene-28-olic acid	C_42_H_66_O_13_	778.4503	fruits	MS, ^13^C-NMR, ^1^H-NMR, DEPT, HMBC, HMQC, NOESY	[19]
**9**	(1R,11*α*)-1,4-Epoxy-11-hydroxy-3,4-secolupane-20(30)-ene-3,28-Dioic acid	C_30_H_46_O_6_	502.3294	fruits	MS, ^13^C-NMR, ^1^H-NMR, DEPT, HMBC, HMQC, NOESY	[19]
**10**	(1R,11*α*,22*α*)-1,4-Epoxy-11,22-hydroxy-3,4-secolupane-20(30)-ene-3,28-dioic acid	C_30_H_46_O_7_	518.3244	fruits	MS, ^13^C-NMR, ^1^H-NMR, DEPT, HMBC, HMQC, NOESY	[19]
**11**	Elesesterpene A	C_30_H_46_O_6_	502.3294	leaves	X-ray, ^13^C-NMR, ^1^H-NMR	[16]
**12**	Elesesterpene B	C_31_H_48_O_5_	500.3502	leaves	X-ray, ^13^C-NMR, ^1^H-NMR	[16]
**13**	Elesesterpene C	C_50_H_80_O_19_	984.5294	leaves	X-ray, ^13^C-NMR, ^1^H-NMR	[16]
**14**	Elesesterpene D	C_49_H_78_O_18_	954.5188	leaves, fruits	HR-MS, X-ray, ^13^C-NMR, ^1^H-NMR	[9,16]
**15**	Elesesterpene E	C_50_H_80_O_18_	968.5345	leaves	X-ray, ^13^C-NMR, ^1^H-NMR	[16]
**16**	Elesesterpene F	C_30_H_44_O_7_	516.3087	leaves	X-ray, ^13^C-NMR, ^1^H-NMR	[16]
**17**	Elesesterpene G	C_29_H_42_O_6_	486.2981	leaves	X-ray, ^13^C-NMR, ^1^H-NMR	[16]
**18**	Elesesterpene H	C_31_H_48_O_7_	532.3400	leaves	X-ray, ^13^C-NMR, ^1^H-NMR	[16]
**19**	Elesesterpene I	C_32_H_50_O_7_	546.3557	leaves	X-ray, ^13^C-NMR, ^1^H-NMR	[16]
**20**	Elesesterpene J	C_30_H_46_O_6_	502.3294	leaves	X-ray, ^13^C-NMR, ^1^H-NMR	[16]
**21**	Elesesterpene K	C_50_H_80_O_20_	1000.5243	leaves	X-ray, ^13^C-NMR, ^1^H-NMR	[16]
**22**	24-Hydroxychiisanoside	C_48_H_74_O_20_	970.4773	leaves	UPLC-Q-TOF-MS	[20]
**23**	Hederacoside D	C_53_H_86_O_22_	1074.5611	leaves	UPLC-Q-TOF-MS	[20]
**24**	Cauloside D	C_53_H_86_O_22_	1074.5611	leaves	UPLC-Q-TOF-MS	[20]
**25**	Nipponoside B	C_54_H_88_O_22_	1088.5767	leaves	UPLC-Q-TOF-MS	[20]
**26**	Saniculoside N	C_55_H_88_O_22_	1100.5767	leaves	UPLC-Q-TOF-MS	[20]
**27**	Guaianin N	C_41_H_66_O_12_	750.4554	leaves	UPLC-Q-TOF-MS	[20]
**28**	Eleutheroside I	C_41_H_66_O_11_	734.4605	leaves	UPLC-Q-TOF-MS	[20]
**29**	Hemsgiganoside B	C_48_H_76_O_19_	956.4981	leaves	UPLC-Q-TOF-MS	[20]
**30**	1-Deoxyisochiisanoside	C_48_H_76_O_19_	956.4981	leaves	UPLC-Q-TOF-MS	[20]
**31**	Ciwujianoside C3	C_53_H_86_O_21_	1058.5662	leaves	UPLC-Q-TOF-MS	[20]
**32**	Anhuienside C	C_53_H_86_O_21_	1058.5662	leaves	UPLC-Q-TOF-MS	[20]
**33**	Ciwujianoside D1	C_55_H_88_O_22_	1100.5767	leaves	UPLC-Q-TOF-MS	[20]
**34**	Ciwujianoside B	C_58_H_92_O_25_	1188.5928	leaves	UPLC-Q-TOF-MS	[20]
**35**	Ciwujianoside D2	C_54_H_84_O_22_	1084.5454	leaves	UPLC-Q-TOF-MS	[20]
**36**	Ciwujianoside E	C_40_H_62_O_11_	718.4292	leaves	UPLC-Q-TOF-MS	[20]
**37**	Ciwujianoside D3	C_55_H_88_O_23_	1116.5716	leaves	UPLC-Q-TOF-MS	[20]
**38**	Alphitolic acid	C_30_H_48_O_4_	472.3553	leaves	NMR, MS	[32]
**39**	11-Deoxyisochiisanoside	C_48_H_76_O_19_	956.4981	leaves	IR, ^13^C-NMR, ^1^H-NMR	[33]
**40**	Isochiisanoside	C_48_H_76_O_19_	956.4981	leaves	IR, ^13^C-NMR, ^1^H-NMR	[33]
**41**	3-Oxo-24-methylenecycloartan	C_31_H_50_O	438.3862	fruits	MS, ^13^C-NMR, ^1^H-NMR	[14]
**42**	Schizandronic acid	C_30_H_46_O_3_	454.3447	fruits	MS, ^13^C-NMR, ^1^H-NMR	[14]
**43**	Isomangiferolic acid	C_32_H_52_O_2_	468.3967	fruits	MS, ^13^C-NMR, ^1^H-NMR	[14]
**44**	Betulin	C_30_H_50_O_2_	442.3811	fruits	MS, ^13^C-NMR, ^1^H-NMR	[14]
**45**	Betulinic acid	C_30_H_48_O_3_	456.3603	fruits	MS, ^13^C-NMR, ^1^H-NMR	[14]
**46**	Eleutheroside K	C_41_H_66_O_11_	734.4605	fruits	MS, ^13^C-NMR, ^1^H-NMR	[14]
**47**	22-*α*-Hydroxychiisanogenin	C_30_H_44_O_6_	500.3138	fruits	MS, ^13^C-NMR, ^1^H-NMR	[14]
**48**	Acanthosessilioside F	C_36_H_54_O_11_	662.3666	fruits	MS, ^13^C-NMR, ^1^H-NMR, DEPT, COSY, NOESY, HSQC, HMBC	[7]
**49**	Acanthosessiligenin II	C_31_H_48_O_6_	516.3451	fruits	MS, ^13^C-NMR, ^1^H-NMR, DEPT, COSY, NOESY, HSQC, HMBC	[7]
**50**	Acanthosessilioside B	C_37_H_58_O_11_	678.3979	fruits	MS, ^13^C-NMR, ^1^H-NMR, DEPT, COSY, NOESY, HSQC, HMBC	[7]
**51**	Acanthosessilioside C	C_37_H_58_O_12_	694.3928	fruits	MS, ^13^C-NMR, ^1^H-NMR, DEPT, COSY, NOESY, HSQC, HMBC	[7]
**52**	Acanthosessilioside E	C_36_H_56_O_11_	664.3823	fruits	MS, ^13^C-NMR, ^1^H-NMR, DEPT, COSY, NOESY, HSQC, HMBC	[7]
**53**	Acanthosessilioside D	C_36_H_56_O_10_	648.3873	fruits	MS, ^13^C-NMR, ^1^H-NMR, DEPT, COSY, NOESY, HSQC, HMBC	[7]
**54**	Acanthosessiligenin I	C_31_H_48_O_5_	500.3502	fruits	MS, ^13^C-NMR, ^1^H-NMR, DEPT, COSY, NOESY, HSQC, HMBC	[7]
**55**	Acanthosessilioside A	C_36_H_56_O_9_	632.3924	fruits	MS, ^13^C-NMR, ^1^H-NMR, DEPT, COSY, NOESY, HSQC, HMBC	[7]
**56**	Sessiloside	C_48_H_76_O_20_	972.4930	leaves, fruits	UPLC-MS, IR, ^13^C-NMR, ^1^H-NMR	[9,33]
**57**	Calenduloside E 6′-methyl ester	C_37_H_58_O_9_	646.4081	fruits	IR, ^13^C-NMR, ^1^H-NMR, COSY, HSQC, HMBC	[34]
**58**	Acanthosessilioside G	C_42_H_66_O_15_	810.4402	fruits	UPLC-MS, ^13^C-NMR, ^1^H-NMR, IR	[9]
**59**	Acanthosessilioside H	C_48_H_76_O_19_	956.4981	fruits	UPLC-MS, ^13^C-NMR, ^1^H-NMR, IR	[9]
**60**	Acanthosessilioside I	C_49_H_78_O_19_	970.5137	fruits	UPLC-MS, ^13^C-NMR, ^1^H-NMR, IR	[9]
**61**	Acanthosessilioside O	C_49_H_78_O_21_	1002.5036	fruits	UPLC-MS, ^13^C-NMR, ^1^H-NMR, IR	[9]
**62**	Acanthosessilioside K	C_51_H_80_O_20_	1012.5243	fruits	UPLC-MS, ^13^C-NMR, ^1^H-NMR, IR	[9]
**63**	Acanthosessilioside L	C_49_H_78_O_19_	970.5137	fruits	UPLC-MS, ^13^C-NMR, ^1^H-NMR, IR	[9]
**64**	Acanthosessilioside M	C_52_H_84_O_20_	1028.5556	fruits	UPLC-MS, ^13^C-NMR, ^1^H-NMR, IR	[9]
**65**	Acanthosessilioside N	C_49_H_78_O_21_	1002.5036	fruits	UPLC-MS, ^13^C-NMR, ^1^H-NMR, IR	[9]
**66**	(2E)-3,7-Dimethylocta-2,6-dienoate-6-O-*α*-L-arabinopyranosyl-(1→6)-*β*-D-glucopyranoside	C_21_H_34_O_11_	462.2101	fruits	IR, HR-MS, ^13^C-NMR, ^1^H-NMR, DEPT, HMBC, HMQC, NOESY	[25]
**67**	(3Z,6E)-3,7-Dimethyl-3,6-octadiene-1,2,8-triol	C_10_H_18_O_3_	186.1256	fruits	IR, HR-MS, ^13^C-NMR, ^1^H-NMR, DEPT, HMBC, HMQC, NOESY	[25]
**68**	(6E)-7-Methyl-3-methylene-6-octene-1,2,8-triol	C_10_H_18_O_3_	186.1256	fruits	IR, HR-MS, ^13^C-NMR, ^1^H-NMR, DEPT, HMBC, [HMQC, NOESY	[25]
**69**	Kenposide A	C_21_H_36_O_10_	448.2308	fruits	IR, HR-MS, ^13^C-NMR, ^1^H-NMR, DEPT, HMBC, HMQC, NOESY	[25]
**70**	Sacranoside B	C_21_H_36_O_10_	448.2308	fruits	IR, HR-MS, ^13^C-NMR, ^1^H-NMR, DEPT, HMBC, HMQC, NOESY	[25]
**71**	1-O-[(S)-Oleuropeyl]-*β*-D-glucopyranose	C_16_H_26_O_8_	346.1628	fruits	IR, HR-MS, ^13^C-NMR, ^1^H-NMR, DEPT, HMBC, HMQC, NOESY	[25]
**72**	Curcumenol	C_15_H_22_O_2_	234.1620	leaves	UPLC-MS	[20]
**73**	Nootkatone	C_15_H_22_O	218.1671	leaves	UPLC-MS	[20]
**74**	Icariside B1	C_19_H_30_O_8_	386.1941	fruits	MS, ^13^C-NMR, ^1^H-NMR	[14]
**75**	Icariside B2	C_19_H_30_O_8_	386.1941	fruits	MS, ^13^C-NMR, ^1^H-NMR	[14]
**76**	(6R,7E,9R)-9-Hydorxy-4,7-megastigmadien-3-one-9-O-*β*-D-apiofuranosyl-(1–6)-*β*-D-glucopyranoside	C_24_H_38_O_11_	502.2414	fruits	MS, ^13^C-NMR, ^1^H-NMR	[14]

IR: Infrared spectroscopy; ^13^C-NMR: Carbon-13 nuclear magnetic resonance spectrometry; ^1^H-NMR: Hydrogen-1 nuclear magnetic resonance spectrometry; HPLC: High-performance liquid chromatography; HPLC-MS: High-performance liquid chromatography-mass spectrometry; UV: Ultraviolet spectrophotometry; IR: Infrared spectroscopy; NMR: Nuclear magnetic resonance spectrometry; HR-MS: High-resolution mass spectrometry; MS: Mass spectrometry; DEPT: Distortionless enhancement by polarization transfer; HMBC: ^1^H Detected heteronuclear multiple bond correlation, HMQC: ^1^H Detected heteronuclear multiple quantum coherence, NOESY: Nuclear overhauser effect spectroscopy; X-ray: X-ray crystallographic analysis; UPLC-MS: Ultra performance liquid chromatography-mass spectrometry; COSY: (Homonuclear chemical shift) correlation spectroscopy; HSQC: Heteronuclear single quantum coherence.

### 2.2. Phenylpropanoids

**Figure 3:**
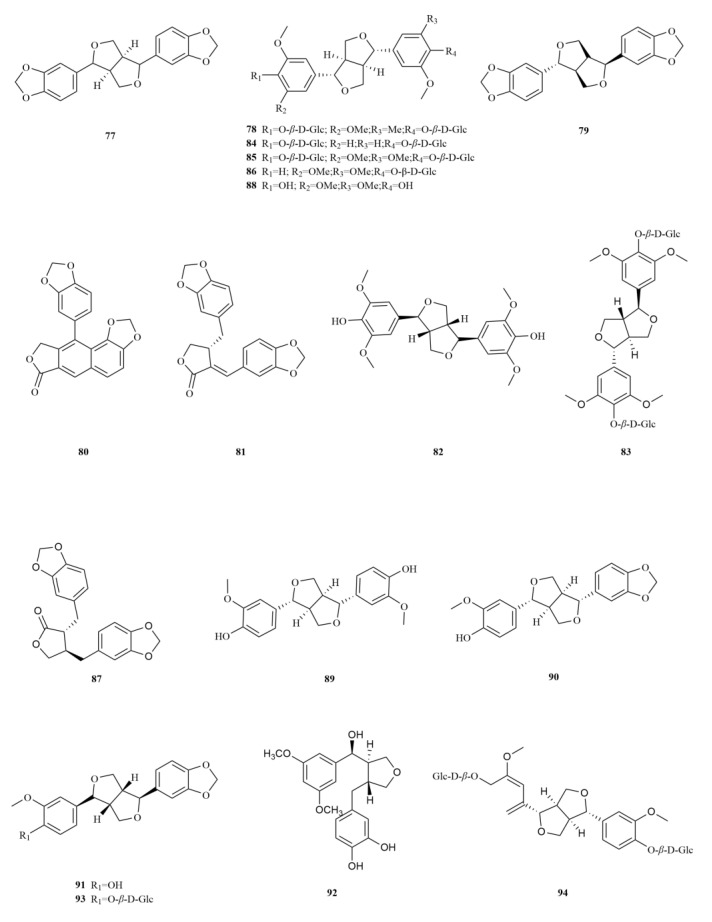

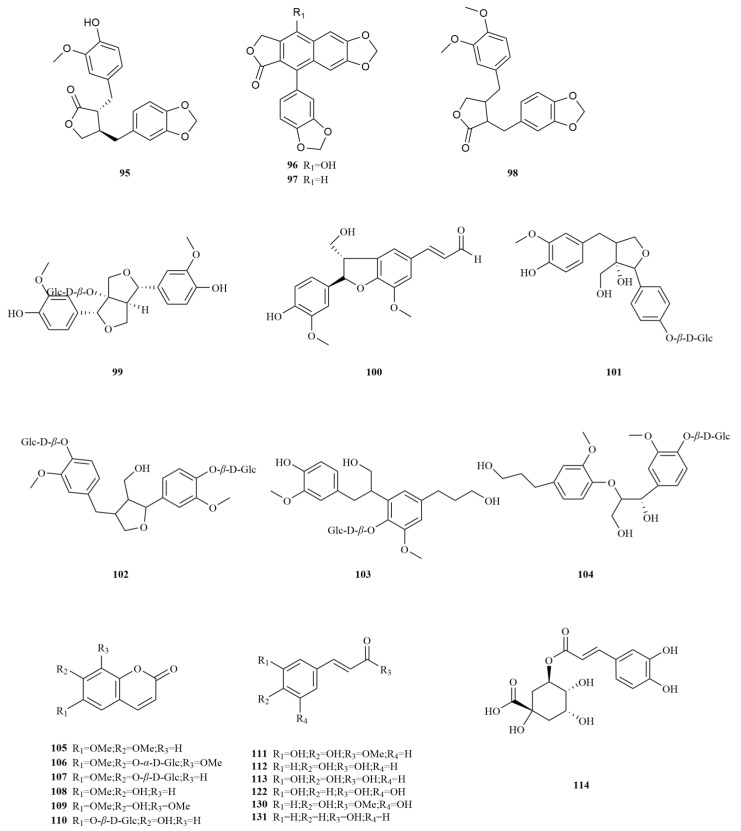

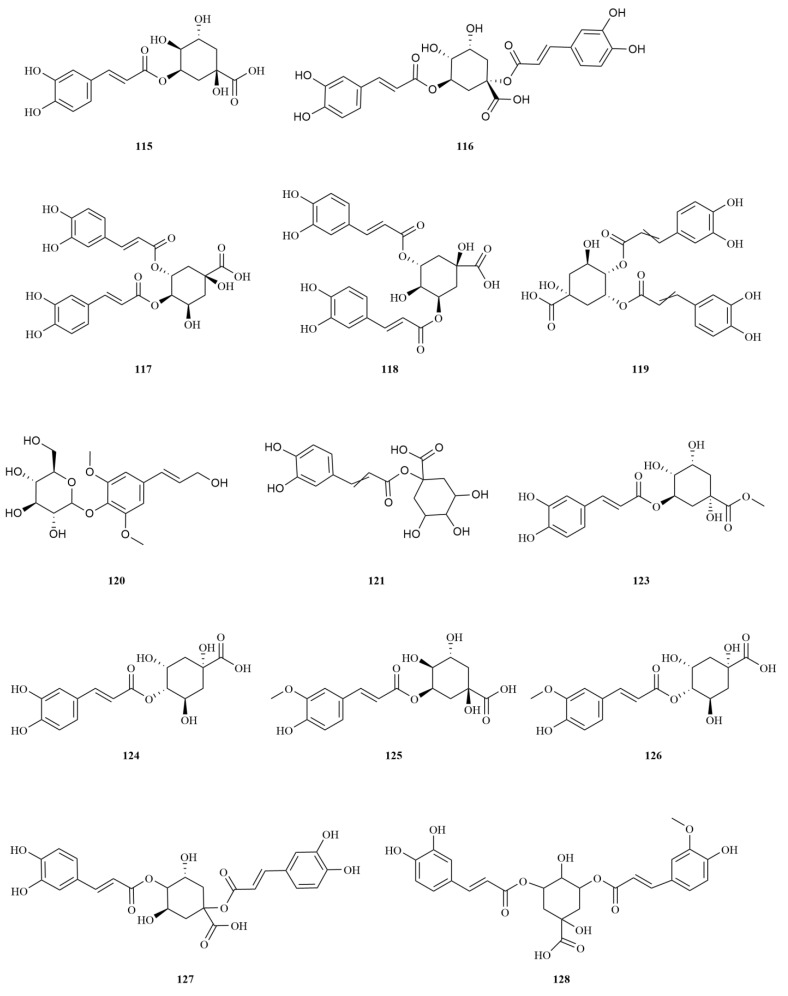

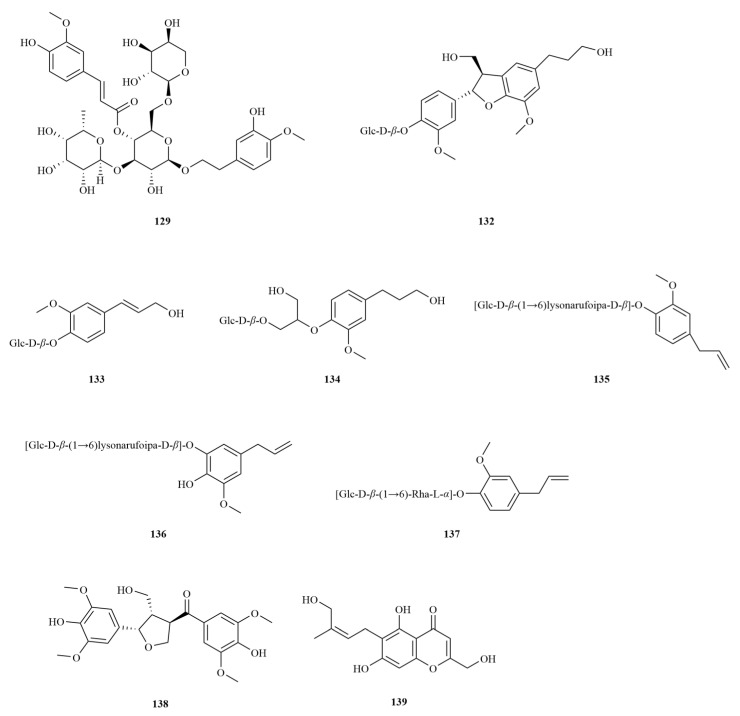
Chemical structures of phenylpropanoids isolated from *Eleutherococcus sessiliflorus.* Chemical structures were drawn using Chemdraw Professional 15.0 software.

*E. sessiliflorus* is rich in phenylpropanoids, with a total of sixty-three compounds identified, including simple phenylpropanoids, lignans, and coumarins (Table 2, Figure 3). A notable discovery in *E. sessiliflorus* was scoparone (lignans), which was isolated for the first time from its fruits, marking a novel finding within the *Eleutherococcus* genus [22]. Scoparone has anti-inflammatory, analgesic, and anti-coagulant effects. Recent research has found that it (500 μM) inhibits breast cancer cell viability through the NF-*κ*B signaling pathway [35].

Starting with the roots of *E. sessiliflorus*, eight phenylpropanoid compounds were successfully isolated from the ethyl acetate fraction of the 70% ethanol extract. Among these compounds, lignans like (+)-episesamin (**79**), helioxanthin (**80**), and (−)-syringaresinol (**82**), alongside caffeic acid methyl ester (**111**) and *p*-hydrocoumaric acid (**112**) were found [36]. It is important to highlight that both (+)-episesamin and caffeic acid methyl ester represent newly discovered compounds within the *Eleutherococcus* genus. Moving on to the fruits, a total of nine lignan compounds were isolated from the ethanol extract. Among them, acanthosessilin A (**92**), a novel lignan, was identified for the first time in the *Eleutherococcus* genus. Additionally, other compounds like (+)-piperitol (**90**), (+)-xanthoxylol (**91**), and simplexoside (**93**) were observed, further enhancing our understanding of the repertoire of lignans in *E. sessiliflorus*. Interestingly, hinokinin (**87**) and (+)-pinoresinol (**89**) (Table 2, Figure 3) were also first discovered within *E. sessiliflorus* [37]. Hinokinin (20 or 40 mg/kg) protects against high-fat diet/streptozotocin-induced cardiac injury in mice by alleviating oxidative stress, inflammation, and apoptosis [38].

A comparative analysis was conducted to investigate the differences in the chemical composition among the roots of *E. sessiliflorus*, *Eleutherococcus nodiflorus*, and *E. senticosus*. Notably, the compounds taiwanin C (**97**) and taiwanin E (**96**) (Table 2, Figure 3) were found to be unique to *E. sessiliflorus*, highlighting its distinct chemical profile. Normal oral cells N28 and oral cancer cells T28 were treated with different concentrations of taiwanin C at 0, 1, 5, 10, 30, and 60 μM. Results showed the therapeutic potential of taiwanin C against arecoline-induced oral cancer and no significant cytotoxicity for normal oral cells [39]. Taiwanin E (0.5, 1, 5, 10, and 20 μM) inhibits cell migration in human lovo colon cancer cells by suppressing MMP-2/9 expression through the p38 MAPK pathway [40]. Moreover, a comparative analysis was carried out to examine the variation in the chemical composition among the roots of *E. sessiliflorus*, *Eleutherococcus nodiflorus*, and *E. senticosus*. It was discovered that fourteen components were common to *E. senticosus* and *E. sessiliflorus*. A comparative analysis was conducted to explore the differences in chemical composition among the roots of *E. sessiliflorus*, *Eleutherococcus nodiflorus*, and *E. senticosus*. Interestingly, it was observed that *E. senticosus* contained various phenolic glycosides that were not detected in both *E. sessiliflorus* and *Eleutherococcus nodiflorus*, suggesting a higher similarity in chemical composition between *E. sessiliflorus* and *Eleutherococcus nodiflorus* [6]. This finding supports considering *E. sessiliflorus* as a potential substitute for *Eleutherococcus nodiflorus.*

To examine the changes in content, a study employed HPLC to simultaneously measure six components in the green and mature fruits of *E. sessiliflorus*. The study observed that as the fruits of *E. sessiliflorus* matured, there was an increasing trend in the content of (−)-pinoresinol-4,4′-di-O-*β*-D-glucopyranoside (**84**), acanthoside D (**85**), acanthoside B (**86**), and scopolin (**107**) (Table 2, Figure 3). This suggests a gradual elevation in the concentration of active compounds during fruit growth, indicating the potential for higher medicinal properties [41]. Another investigation focused on determining the content of eleutheroside E and eleutheroside B in different parts (roots, stems, leaves, and fruits) of *E. sessiliflorus* through a 50% methanol extract. The results revealed that the fruits and stems had the highest contents of eleutheroside B and E. Importantly, no cytotoxic effects were observed on the normal cell line (DC2.4), and the roots extract exhibited a 23% inhibition rate on the stomach cancer cell line (SNU-719), highlighting its potential as a health food [42]. Furthermore, since the initial discovery of scoparone (**105**), a coumarin in *E. sessiliflorus* fruits, subsequent research has identified six coumarins from various parts of the plant, including the roots, stems, fruits, and leaves. Among these, seopoletin (**108**) and isofraxidin (**109**) (Table 2, Figure 3) have been identified in *E. sessiliflorus*, with contents of 30.5 μg/g and 7.90 μg/g, respectively [43]. Isofraxidin (3 and 15 mg/kg) possesses significant analgesic and anti-inflammatory activities that may be mediated by regulating pro-inflammatory cytokines, TNF-*α* and the phosphorylation of p38 and ERK1/2 [44].

Coumarins exhibit a diverse range of pharmacological effects, including but not limited to anti-inflammatory, anti-coagulant, antimicrobial, anticancer, antihypertensive, antituberculous, anticonvulsant, and antihyperglycemic activities. Additionally, they possess antioxidant and neuroprotective properties [45]. Notably, scoparone demonstrates anti-inflammatory, antioxidant, anti-apoptotic, anti-fibrotic, and lipid-lowering properties, making it a compound with multiple potential therapeutic benefits [46]. On the other hand, esculin (**110**), another coumarin found in *E. sessiliflorus*, exhibits anti-diabetic effects, promoting improvements in pancreatic damage, enhanced insulin secretion, and glucose homeostasis. In addition, esculin, a coumarin compound found in *E. sessiliflorus*, has several therapeutic properties, including anticancer, antibacterial, antiviral, neuroprotective, antithrombotic, and ophthalmic effects [47] (Table 2, Figure 3).

Simple phenylpropanoids in *E. sessiliflorus* have been extensively studied in fruits and roots (Table 2, Figure 3). Two specific phenylpropanoids, *p*-hydroxycoumaric acid (**112**) and caffeic acid (**113**), were identified in the 70% ethanol extract of the fruits [48]. Both *p*-Hydroxycoumaric acid and caffeic acid showed good antioxidant capacities [49]. Furthermore, 3,5-dihydroxycinnamic acid (**122**) was also isolated from the same extract, marking its first-time identification in *E. sessiliflorus*. This compound displayed potent ABTS and DPPH radical scavenging abilities, indicating its strong antioxidant properties [50]. Additionally, HPLC analysis was conducted to determine the content of seven organic acids (caffeic acid, chlorogenic acid, neochlorogenic acid, 1,3-dicaffeoylquinic acid, isochlorogenic acid A-C (**113**–**119**) in the root at different growth stages ranging from four to eight years. The content of each organic acid increased with the age of the plant, with the 8th year showing the highest levels. Notably, chlorogenic acid was the most abundant [51].

**Table 2:** Phenylpropanoids isolated from *Eleutherococcus sessiliflorus.*

NO.	Name	Formula	Exact Theoretical Molecular Weight	Source	Characterization method	Refs.
**77**	(−)-Sesamin	C_20_H_18_O_6_	354.1103	fruits, roots	IR, ^13^C-NMR, ^1^H-NMR, MS	[22,36]
**78**	Liriodendrin	C_34_H_46_O_18_	742.2684	stem barks	Unspecified	[52]
**79**	(+)-Episesamin	C_20_H_18_O_6_	354.1103	roots	^13^C-NMR, ^1^H-NMR, MS	[36]
**80**	Helioxanthin	C_20_H_12_O_6_	348.0634	roots	^13^C-NMR, ^1^H-NMR, MS	[36]
**81**	Savinin	C_20_H_16_O_6_	352.0947	roots, leaves	UPLC-MS, ^13^C-NMR, ^1^H-NMR, MS	[20,36]
**82**	(−)-Syringaresinol	C_22_H_26_O_8_	418.1628	roots	^13^C-NMR, ^1^H-NMR, MS	[36]
**83**	Eleutheroside E	C_34_H_46_O_18_	742.2684	roots, stems, fruits	HPLC	[42]
**84**	(−)-Pinoresinol-4,4′-di-O-*β*-D-glucopyranoside	C_32_H_42_O_16_	682.2473	fruits	HPLC	[41]
**85**	Acanthoside D	C_34_H_46_O_18_	742.2684	fruits, roots, root barks	UPLC- MS, HPLC, ^13^C-NMR, ^1^H-NMR	[6,41,53]
**86**	Acanthoside B	C_28_H_36_O_13_	580.2156	fruits, roots, root barks	UPLC- MS, HPLC, ^13^C-NMR, ^1^H-NMR	[6,41,53]
**87**	Hinokinin	C_20_H_18_O_6_	354.1103	fruits	HR-MS, ^13^C-NMR, ^1^H-NMR, DEPT, COSY, HSQC, HMBC, NOESY, IR	[37]
**88**	(+)-Syringaresinol	C_22_H_26_O_8_	418.1628	fruits	HR-EI-MS, ^13^C-NMR, ^1^H-NMR, DEPT, COSY, HSQC, HMBC, NOESY, IR	[37]
**89**	(+)-Pinoresinol	C_20_H_22_O_6_	358.1416	fruits, roots	^13^C-NMR, ^1^H-NMR, MS, HR-MS, DEPT, COSY, HSQC, HMBC, NOESY, IR	[36,37]
**90**	(+)-Piperitol	C_20_H_20_O_6_	356.1260	fruits	HR-MS, ^13^C-NMR, ^1^H-NMR, DEPT, COSY, HSQC, HMBC, NOESY, IR	[37]
**91**	(+)-Xanthoxylol	C_20_H_20_O_6_	356.1260	fruits	HR-MS, ^13^C-NMR, ^1^H-NMR, DEPT, COSY, HSQC, HMBC, NOESY, IR	[37]
**92**	Acanthosessilin A	C_20_H_24_O_6_	360.1573	fruits	HR-EI-MS, ^13^C-NMR, ^1^H-NMR, DEPT, COSY, HSQC, HMBC, NOESY, IR	[37]
**93**	Simplexoside	C_26_H_30_O_11_	518.1788	fruits, roots	UPLC- MS, HR-MS, ^13^C-NMR, ^1^H-NMR, DEPT, COSY, HSQC, HMBC, NOESY, IR	[6,37]
**94**	(+)-Pinoresinol di-O-*β*-D-glucopyranoside	C_32_H_42_O_16_	682.2473	roots	UPLC-MS	[6]
**95**	Pluviatolide	C_20_H_20_O_6_	356.1260	roots	UPLC-MS	[6]
**96**	Taiwanin E	C_20_H_12_O_7_	364.0583	roots	UPLC-MS	[6]
**97**	Taiwanin C	C_20_H_12_O_6_	348.0634	roots	UPLC-MS	[6]
**98**	3-(3,4-Dimethoxybenzyl)-2-(3,4-methylenedioxybenzyl) butyrolactone	C_21_H_22_O_6_	370.1416	roots	UPLC-MS	[6]
**99**	(+)-l-Hydroxypinoresinol-l-O-β-D-glucoside	C_26_H_32_O_12_	536.1894	fruits	MS, ^13^C-NMR, ^1^H-NMR	[14]
**100**	Balanophonin	C_20_H_20_O_6_	356.1260	fruits	MS, ^13^C-NMR, ^1^H-NMR	[14]
**101**	Berchemol-4’-O-β-D-glucoside	C_26_H_34O12_	538.2050	fruits	MS, ^13^C-NMR, ^1^H-NMR	[14]
**102**	Lariciresinol-4,4’-di-O-β-D-glucopyranoside	C_32_H_34_O_11_	594.2101	fruits	MS, ^13^C-NMR, ^1^H-NMR	[14]
**103**	Icariside E3	C_26_H_36_O_11_	524.2258	fruits	MS, ^13^C-NMR, ^1^H-NMR	[14]
**104**	(7S,8R)-Erythro-7,9,9’-trihydroxy-3,3’-dimethoxy-8-O-4’-neolignan-4-O-*β*-D-glucopyranosideerythro	C_26_H_36_O_12_	540.2207	fruits	MS, ^13^C-NMR, ^1^H-NMR	[14]
**105**	Scoparone	C_11_H_10_O_4_	206.0579	fruits	IR, ^13^C-NMR, ^1^H-NMR	[22]
**106**	Isofraxidin-7-O-*α*-D-glucoside	C_13_H_12_O_5_	248.0685	stems	IR, ^13^C-NMR, ^1^H-NMR	[54]
**107**	Scopolin	C_16_H_18_O_9_	354.0951	fruits	HPLC	[41]
**108**	Scopoletin	C_10_H_8_O_4_	192.0423	fruits	HPLC-MS, ^13^C-NMR, ^1^H-NMR	[30]
**109**	Isofraxidin	C_11_H_10_O_5_	222.0528	roots, fruits, leaves	UPLC-MS, HPLC-MS, HPLC	[20,29,43]
**110**	Esculin	C_15_H_16_O_9_	340.0794	leaves	UPLC-MS	[20]
**111**	Caffeic acid methyl ester	C_10_H_10_O_4_	194.0579	roots	^13^C-NMR, ^1^H-NMR, MS	[36]
**112**	*p*-Hydrocoumaric acid	C_9_H_10_O_3_	166.0630	roots, fruits	^13^C-NMR, ^1^H-NMR, MS	[36,48]
**113**	Caffeic acid	C_9_H_8_O_4_	180.0423	roots, fruits	^13^C-NMR, ^1^H-NMR, MS, HPLC	[48,51]
**114**	Chlorogenic acid	C_16_H_18_O_9_	354.0951	roots	HPLC	[51]
**115**	Neochlorogenic acid	C_16_H_18_O_9_	354.0951	roots	HPLC	[51]
**116**	1,3-Dicaffeoylquinic acid	C_25_H_24_O_12_	516.1268	roots	HPLC	[51]
**117**	Isochlorogenic acid B	C_25_H_24_O_12_	516.1268	roots	HPLC	[51]
**118**	Isochlorogenic acid A	C_25_H_24_O_12_	516.1268	roots	HPLC	[51]
**119**	Isochlorogenic acid C	C_25_H_24_O_12_	516.1268	roots	HPLC	[51]
**120**	Syringin	C_17_H_24_O_9_	372.1420	root barks, stems	HPLC	[42,55]
**121**	Caffeoylquinic acid	C_16_H_18_O_9_	354.0951	roots, leaves	UPLC-MS, UPLC-MS	[6,20]
**122**	3,5-Dihydroxycinnamic acid	C_9_H_8_O_4_	180.0423	fruits	NMR, MS, IR	[50]
**123**	Chlorogenic acid methyl ester	C_17_H_20_O_9_	368.1107	root barks	^13^C-NMR, ^1^H-NMR	[53]
**124**	Cryptochlorogenic acid	C_16_H_18_O_9_	354.0951	leaves	UPLC-MS	[20]
**125**	3-Feruloylquinic acid	C_17_H_20_O_9_	368.1107	leaves	UPLC-MS	[20]
**126**	4-Feruloylquinic acid	C_17_H_20_O_9_	368.1107	leaves	UPLC-MS	[20]
**127**	1,4-Dicaffeoylquinic acid	C_25_H_24_O_12_	516.1268	leaves	UPLC-MS	[20]
**128**	3-Feruloyl-5-caffeoylquinic acid	C_26_H_26_O_12_	530.1424	leaves	UPLC-MS	[20]
**129**	Angoroside C	C_36_H_48_O_19_	784.2790	leaves	UPLC-MS	[20]
**130**	Ferulic acid	C_10_H_10_O_4_	194.0579	leaves, roots	UPLC-MS, HPLC-MS	[20,29]
**131**	Cinnamic acid	C_9_H_8_O_2_	148.0524	roots	HPLC-MS	[29]
**132**	(7S,8R) Dihydrodehydrodiconiferyl alcohol 4-O-β-D-glucopyranoside	C_26_H_34_O_11_	522.2101	fruits	MS, ^13^C-NMR, ^1^H-NMR	[14]
**133**	Coniferin	C_16_H_22_O_8_	342.1315	fruits	MS, ^13^C-NMR, ^1^H-NMR	[14]
**134**	4-O-(2-O-β-D-Glucopyranosyl-1-hydroxymethylethly)-dihydroconiferyl alcohol	C_19_H_30_O_10_	418.1839	fruits	MS, ^13^C-NMR, ^1^H-NMR	[14]
**135**	1-Allyl-3-methoxyhenyl-6-O-β-D-apiofuranosyl-(1′′-6′)-β-D-glucopyranoside	C_21_H_30_O_11_	458.1788	fruits	MS, ^13^C-NMR, ^1^H-NMR	[14]
**136**	Hovetrichoside G	C_21_H_30_O_12_	474.1737	fruits	MS, ^13^C-NMR, ^1^H-NMR	[14]
**137**	Eugenyl β-rutinoside	C_22_H_32_O_11_	472.1945	fruits	MS, ^13^C-NMR, ^1^H-NMR	[14]
**138**	Ciwujiatone	C_22_H_26_O_9_	434.1577	root barks	^13^C-NMR, ^1^H-NMR	[53]
**139**	Cnidimol D	C_15_H_16_O_6_	292.0947	leaves	UPLC-MS	[20]

IR: Infrared spectroscopy; ^13^C-NMR: Carbon-13 nuclear magnetic resonance spectrometry; ^1^H-NMR: Hydrogen-1 nuclear magnetic resonance spectrometry; MS: Mass spectrometry; UPLC-MS: Ultra performance liquid chromatography-mass spectrometry; HPLC: High-performance liquid chromatography; HR-MS: High-resolution mass spectrometry; DEPT: Distortionless enhancement by polarization transfer; COSY: (Homonuclear chemical shift) correlation spectroscopy; HMBC: ^1^H Detected heteronuclear multiple bond correlation, HMQC: ^1^H Detected heteronuclear multiple quantum coherence, NOESY: Nuclear overhauser effect spectroscopy; HPLC-MS: High-performance liquid chromatography-mass spectrometry.

### 2.3. Flavonoids

**Figure 4:**
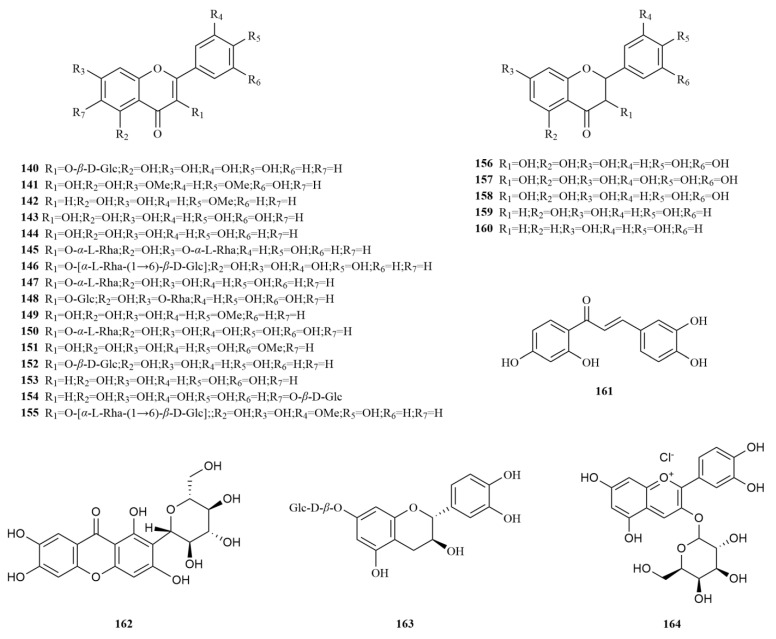
Chemical structures of flavonoids isolated from *Eleutherococcus sessiliflorus.* Chemical structures were drawn using ChemDraw Professional 15.0 software.

To date, twenty-five flavonoids have been identified from *E. sessiliflorus* (Table 3, Figure 4). These include three flavones, thirteen flavonols, two flavanones, three flavanonols, one chalcone (butein, (**162**)), one biphenylketone (mangiferin, (**163**)), one flavan-3-ol (catechin-7-O-*β*-glucopyranoside, (**164**)), and one anthocyanin (cyanidin 3-xylosyl-galactoside, (**165**)) [14,20,32,56]. Butein is a promising anticancer molecule, and its major modes of action in different cancer cells are apoptosis and interference with cell cycle [57]. Mangiferin (40, 80, and 120 mg/kg) decreased macrophage phagocytosis but increased NK cell activities in vivo. Meanwhile, it increased the survival rate of leukemia mice in vivo [58]. Six flavonoids have been isolated and purified from the 70% extract of *E. sessiliflorus* leaves. Among them, dihydromyricetin (**158**), taxifolin (**159**), and butein (**161**) were identified for the first time within the *Eleutherococcus* genus. Furthermore, researchers confirmed that these compounds exhibited relatively weak cytotoxic activity against the cell line A549 (IC_50_ < 88.2 μM), suggesting their potential as advantageous candidates for anticancer drugs [32].

*E. sessiliflorus* is recognized as a novel functional food with a rich content of protein, fiber, and minerals within its fruits. Presently, *E. sessiliflorus* is being cultivated in Poland and has gained appreciation from vegetarian enthusiasts [59]. Additionally, an analysis of flavonoid content was conducted across four *Eleutherococcus* species, namely *Eleutherococcus henryi, Eleutherococcus koreanum, E. senticosus* and *E. sessiliflorus*. Notably, both the roots and stems and the fruits of *E. senticosus* and *E. sessiliflorus* exhibit elevated levels of total flavonoids. Moreover, hyperin is the most abundant flavonoid within *E. sessiliflorus* [60,61]. Hyperin has proven effective in various domains, such as anti-inflammatory, antibacterial, antiviral, neuroprotective, antidepressant, and organ-protective properties. Its broad application in the field of anti-tumor treatments is particularly noteworthy, showing efficacy against lung cancer, cervical cancer, gastric cancer, colorectal cancer, pancreatic cancer, breast cancer, and ovarian cancer [62]. Therefore, the flavonoid-rich *E. sessiliflorus* presents a promising avenue in the domains of medicinal natural products, dietary supplements, and beverages, serving as a potential source of agricultural and industrial innovation.

**Table 3:** Flavonoids isolated from *Eleutherococcus sessiliflorus.*

NO.	Name	Formula	Exact Theoretical Molecular Weight	Source	Characterization Method	Refs.
**140**	Hyperin	C_21_H_20_O_12_	464.0955	fruits, roots	IR, ^13^C-NMR, ^1^H-NMR, HPLC-MS	[22,29]
**141**	Ombuin	C_17_H_14_O_7_	330.0740	stems	IR, ^13^C-NMR, ^1^H-NMR	[54]
**142**	Acacetin	C_16_H_12_O_5_	284.0685	stems	IR, ^13^C-NMR, ^1^H-NMR	[54]
**143**	Quercetin	C_15_H_10_O_7_	302.0427	stems, fruits, roots, leaves	HPLC-MS, MS, IR, ^13^C-NMR, ^1^H-NMR, HPLC	[29,32,54,61]
**144**	Kaempferol	C_15_H_10_O_6_	286.0477	stems, roots,	HPLC-MS, IR, ^13^C-NMR, ^1^H-NMR	[29,54]
**145**	Kaempferitrin	C_27_H_30_O_14_	578.1636	stems	IR, ^13^C-NMR, ^1^H-NMR	[54]
**146**	Rutin	C_27_H_30_O_16_	610.1534	stems, roots, fruits	HPLC	[60,61]
**147**	Afzelin	C_21_H_20_O_10_	432.1056	stems, roots, fruits	HPLC	[60,61]
**148**	Antoside	C_29_H_36_O_15_	624.2054	roots	UPLC-MS	[6]
**149**	Kaempferide	C_16_H_12_O_6_	300.0634	fruits	HPLC	[63]
**150**	Myricitrin	C_21_H_20_O_12_	464.0955	leaves	UPLC-MS	[20]
**151**	Isorhamnetin	C_16_H_12_O_7_	316.0583	leaves	UPLC-MS	[20]
**152**	Astragalin	C_21_H_20_O_11_	448.1006	leaves	UPLC-MS	[20]
**153**	Luteolin	C_15_H_10_O_6_	286.0477	roots	HPLC-MS	[29]
**154**	Isooirenitn	C_21_H_20_O_11_	448.1006	fruits	^13^C-NMR, ^1^H-NMR	[14]
**155**	Isorhamnetin-3-O-rutinoside	C_28_H_32_O_16_	624.1690	fruits	^13^C-NMR, ^1^H-NMR	[14]
**156**	Catechin	C_15_H_14_O_6_	290.0790	leaves, roots	UPLC-MS, HPLC-MS	[20,29]
**157**	Dihydromyricetin	C_15_H_12_O_8_	320.0532	leaves	^13^C-NMR, ^1^H-NMR, MS	[32]
**158**	Taxifolin	C_15_H_12_O_7_	304.0583	leaves	^13^C-NMR, ^1^H-NMR, MS	[32]
**159**	Naringenin	C_15_H_12_O_5_	272.0685	leaves	^13^C-NMR, ^1^H-NMR, MS	[32]
**160**	Liquiritigenin	C_15_H_12_O_4_	256.0736	leaves	^13^C-NMR, ^1^H-NMR, MS	[32]
**161**	Butein	C_15_H_12_O_5_	272.0685	leaves	^13^C-NMR, ^1^H-NMR, MS	[32]
**162**	Mangiferin	C_19_H_18_O_11_	422.0849	leaves	UPLC-MS	[20]
**163**	Catechin-7-O-*β*-glucopyranoside	C_21_H_24_O_11_	452.1319	fruits	^13^C-NMR, ^1^H-NMR	[14]
**164**	Cyanidin-3-xylosyl-galactoside	C_21_H_21_ClO_11_	484.0772	fruits	^13^C-NMR, ^1^H-NMR	[56]

IR: Infrared spectroscopy; ^13^C-NMR: Carbon-13 nuclear magnetic resonance spectrometry; ^1^H-NMR: Hydrogen-1 nuclear magnetic resonance spectrometry; MS: Mass spectrometry; HPLC-MS: High-performance liquid chromatography-mass spectrometry; HPLC: High-performance liquid chromatography; UPLC-MS: Ultra performance liquid chromatography-mass spectrometry.

### 2.4. Volatile Oils

**Figure 5:**
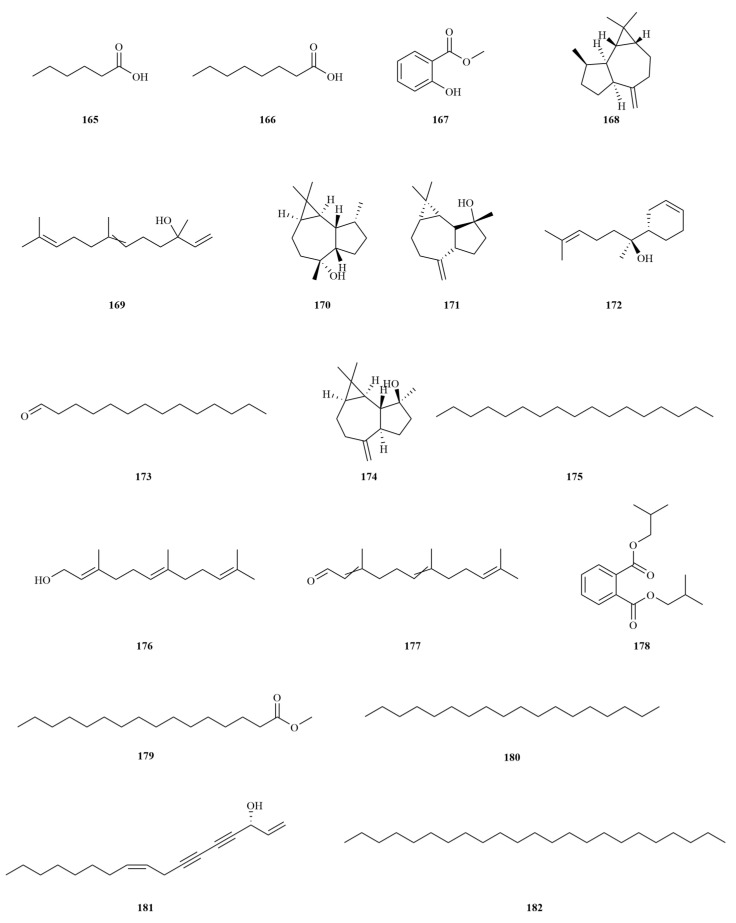

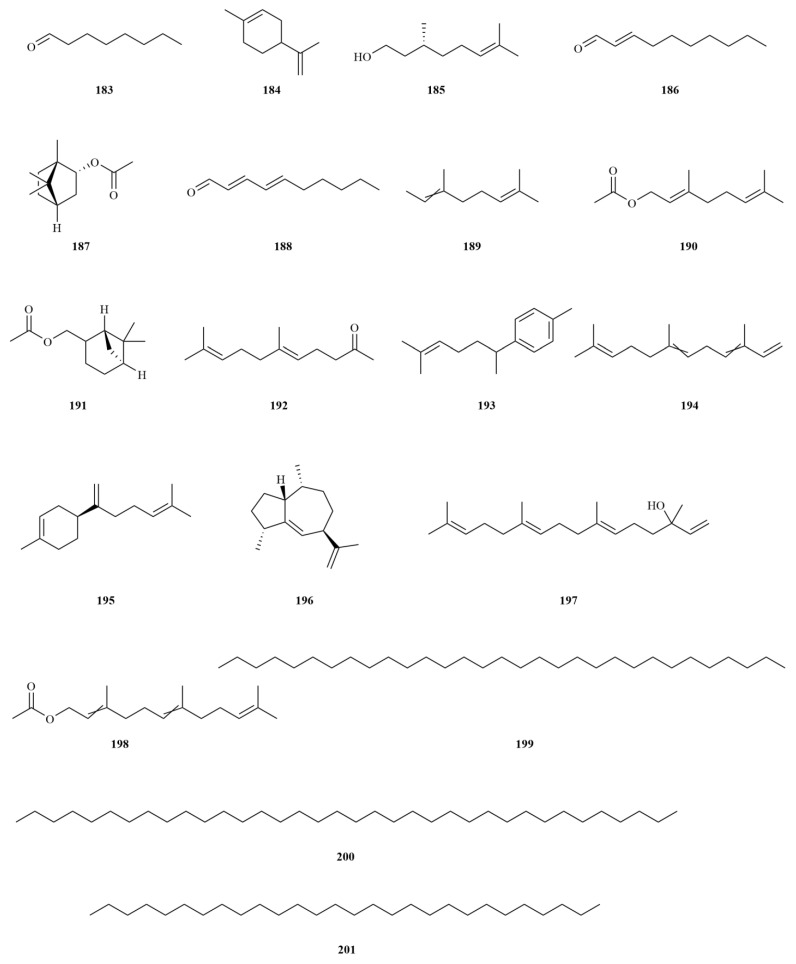

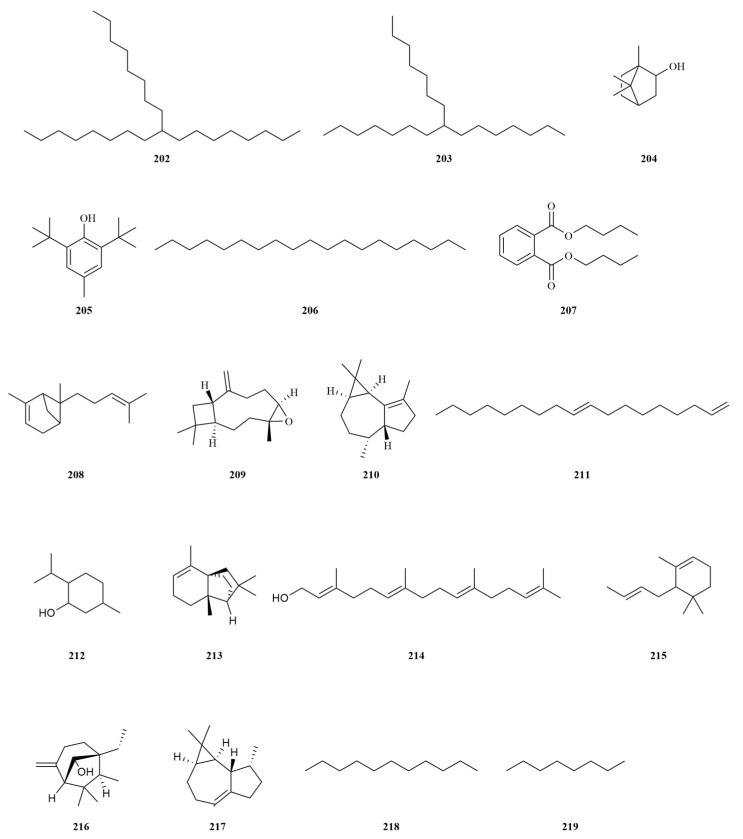
Chemical structures of volatile oils isolated from *Eleutherococcus sessiliflorus.* Chemical structures were drawn using ChemDraw Professional 15.0 software.

Regarding volatile oils, the volatile oils of *E. sessiliflorus* are well-known for their anti-inflammatory and stomach-invigorating properties. Traditional medicine has utilized these oils to treat ailments such as rheumatism and sprains. Not only are these volatile oils beneficial for medicinal purposes, but they can also be used for blending various kinds of perfume. Extensive research has identified fifty-five components from volatile oils, the majority being terpenoids and their derivatives. Among these components, farnesol (**176**) (Table 4, Figure 5) has been found to have the highest content [64,65]. Determination of the volatile oil content is crucial for optimizing extraction techniques and refining processes. Previous research serves as a valuable reference for the development and utilization of volatile oils from *E. sessiliflorus* [59,66].

### 2.5. Organic Acids (Esters)

**Figure 6:**
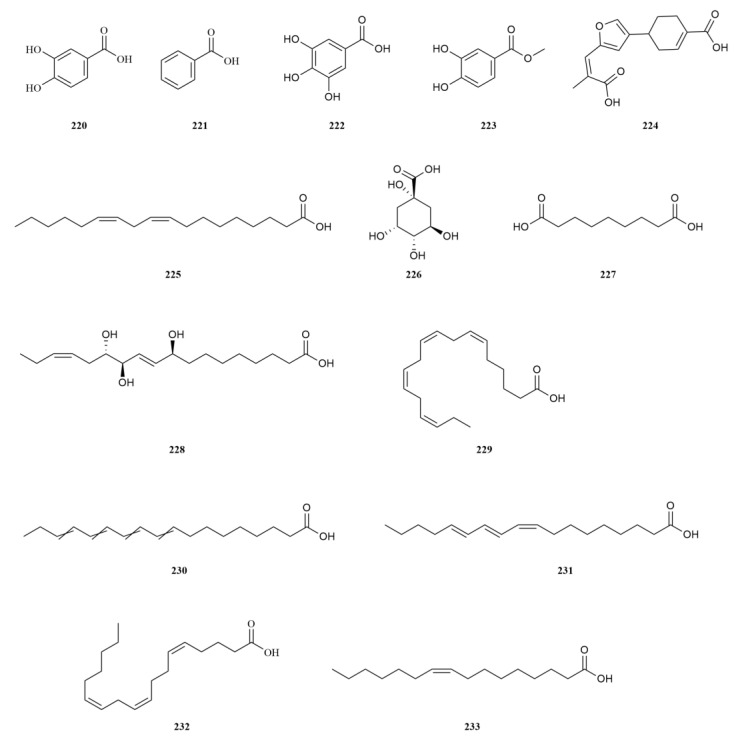

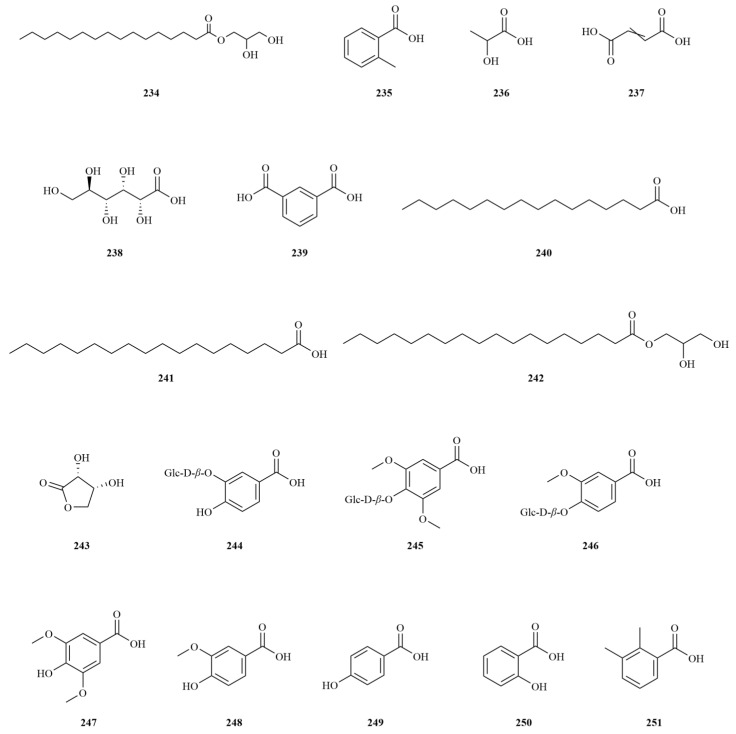
Chemical structures of organic acids (esters) isolated from *Eleutherococcus sessiliflorus.* Chemical structures were drawn using Chemdraw Professional 15.0 software.

A comprehensive analysis has identified thirty-two organic acids (esters) in *E. sessiliflorus* (Table 5, Figure 6). These natural organic acids possess significant antioxidant and anti-inflammatory properties and immunomodulatory effects. The content of organic acids may vary depending on geographic location or plant parts. Utilizing these variations can serve as a basis for evaluating the quality of *E. sessiliflorus* [68]. Furthermore, organic acids, important secondary metabolites, are often used as evaluative indicators to explore differences among various plants. In a comparative analysis conducted on the chemical composition and sedative-hypnotic activity of the leaves of *E. sessiliflorus* and *E. senticosus*, various organic acids (esters) were identified in both plants [20].

To gain insights into the primary metabolites of the leaves, a metabolomic analysis of *E. senticosus* and *E. sessiliflorus* was conducted using gas chromatography-mass spectrometry technology. This analysis included components such as amino acids, organic acids, and fatty acids. Interestingly, the study revealed minimal differences in the composition changes between the leaves of both plants across different periods from May to October. The accumulation of organic acids predominantly occurred during the vigorous growth and senescence phases of the leaves. The above findings provide a reliable basis for dynamically detecting leaf changes using organic acids [67].

### 2.6. Nitrogenous Compounds

**Figure 7:**
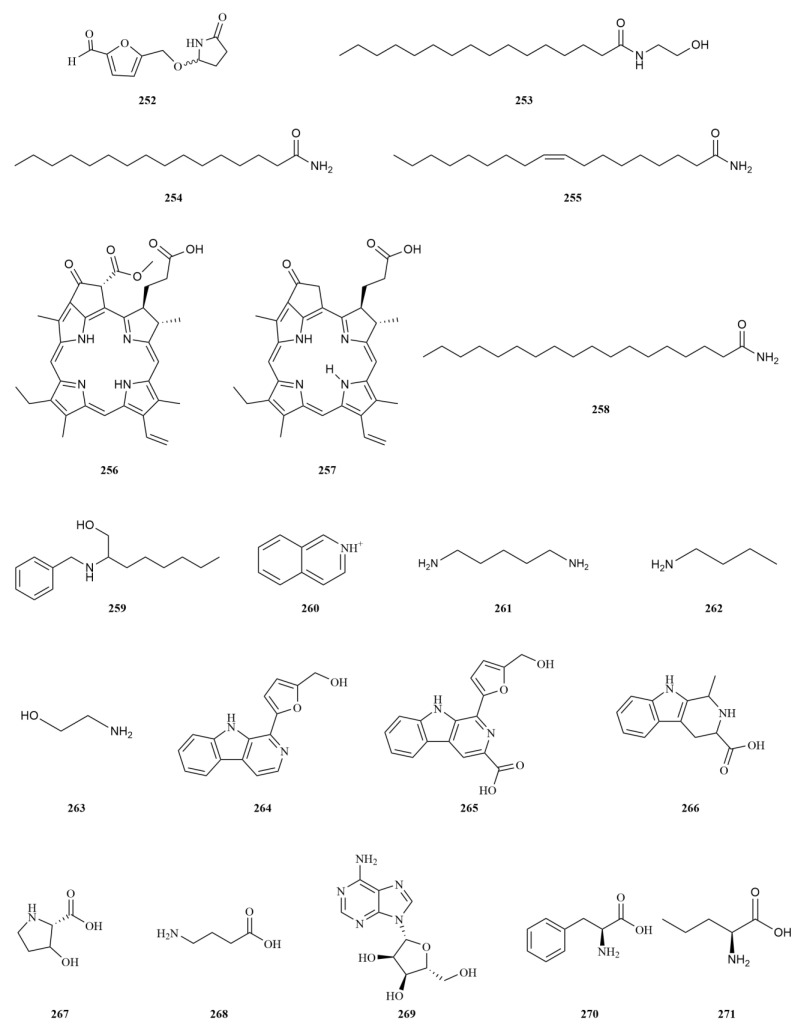
Chemical structures of nitrogenous compounds isolated from *Eleutherococcus sessiliflorus.* Chemical structures were drawn using Chemdraw Professional 15.0 software.

Nitrogenous compounds are vital molecules widely distributed in nature and hold significant biological importance. Many organic compounds containing nitrogen exhibit notable biological activities, including alkaloids and amino acids. In the case of *E. sessiliflorus*, twenty nitrogenous compounds have been identified, with the majority being found in the leaves (Table 6, Figure 7). The discovery of sessiline (**252**) in the fruits has expanded our understanding of nitrogenous compounds in *E. sessiliflorus* [70]. Advanced techniques such as ultra-performance liquid chromatography-mass spectrometry have been employed to identify eight nitrogenous compounds [20]. Additionally, four alkaloids have been identified, namely perlolyrine (**264**), flazine (**265**), 1-methyl-1,2,3,4-tetrahydro-*β*-carboline-3-carboxylic acid (**266**), and adenosine (**269**) [14]. Perlolyrine (20 mg/kg) has a strong anti-platelet effect and a certain degree of antithrombotic effect [71]. Adenosine (10 mg/L in distilled water) induces the activation of AMPK in skeletal muscle and mitigates insulin resistance in mice with high-fat diet-induced diabetes [72].

One noteworthy nitrogenous compound is palmitoylethanolamide (**253**), an endogenous mediator. It has demonstrated favorable tolerability and lacks adverse effects on the body. Palmitoylethanolamide exhibits a broad spectrum of effects, including anti-inflammatory, analgesic, antibacterial, immunomodulatory, and neuroprotective properties [73]. Another compound of interest is oleamide (**255**), which has shown efficacy in sleep enhancement, temperature regulation, and analgesia [74]. However, research on the amino acids present in *E. sessiliflorus* has been limited. Only five amino acids have been identified in this plant, namely ethanolamine (**263**), 3-hydroxy-L-proline (**267**), *γ*-aminobutyric acid (**268**), phenylalanine (**270**), and L-norvaline (**271**) [14,20,67,75]. L-Norvaline (250 mg/L in animals’ water) reverses cognitive decline and synaptic loss in a murine model of Alzheimer’s disease [76].

**Table 6:** Nitrogenous compounds isolated from *Eleutherococcus sessiliflorus.*

NO.	Name	Formula	Exact Theoretical Molecular Weight	Source	Characterization Method	Refs.
**252**	Sessiline	C_10_H_11_NO_4_	209.0688	fruits	IR, ^13^C-NMR, ^1^H-NMR, COSY	[70]
**253**	Palmitoylethanolamide	C_18_H_37_NO_2_	299.2824	leaves	UPLC-MS	[20]
**254**	Hexadecanamide	C_16_H_33_NO	255.2562	leaves	UPLC-MS	[20]
**255**	Oleamide	C_18_H_35_NO	281.2719	leaves	UPLC-MS	[20]
**256**	Pheophorbide A	C_35_H_36_N_4_O_5_	592.2686	leaves	UPLC-MS	[20]
**257**	Pyropheophorbide A	C_33_H_34_N_4_O_3_	534.2631	leaves	UPLC-MS	[20]
**258**	Stearamide	C_18_H_37_NO	283.2875	leaves	UPLC-MS	[20]
**259**	L-2-Benzylaminooctanol	C_15_H_25_NO	235.1936	leaves	GC-MS	[67]
**260**	Isoquinolinium	C_9_H_8_N^+^	130.0651	leaves	GC-MS	[67]
**261**	Cadaverine	C_5_H_14_N_2_	102.1157	leaves	GC-MS	[67]
**262**	*n*-Butylamine	C_4_H_11_N	73.0891	leaves	GC-MS	[67]
**263**	Ethanolamine	C_2_H_7_NO	61.0528	leaves	GC-MS	[67]
**264**	Perlolyrine	C_16_H_12_N_2_O_2_	264.0899	fruits	MS, ^13^C-NMR, ^1^H-NMR	[14]
**265**	Flazine	C_17_H_12_N_2_O_4_	308.0797	fruits	MS, ^13^C-NMR, ^1^H-NMR	[14]
**266**	1-Methyl-1,2,3,4-tetrahydro-*β*-carboline-3-carboxylic acid	C_13_H_14_N_2_O_2_	230.1055	fruits	MS, ^13^C-NMR, ^1^H-NMR	[14]
**267**	3-Hydroxy-L-proline	C_5_H_9_O_3_N	131.0582	fruits	MS, ^13^C-NMR, ^1^H-NMR	[14]
**268**	*γ*-Aminobutyric acid	C_4_H_9_NO_2_	103.0633	aerial part	HPLC	[75]
**269**	Adenosine	C_10_H_13_N_5_O_4_	267.0968	leaves, fruits	MS, ^13^C-NMR, ^1^H-NMR, UPLC-MS	[14,20]
**270**	Phenylalanine	C_9_H_11_NO_2_	165.0790	leaves	UPLC-MS	[20]
**271**	L-Norvaline	C_5_H_11_NO_2_	117.0790	leaves	GC-MS	[67]

IR: Infrared spectroscopy; ^13^C-NMR: Carbon-13 nuclear magnetic resonance spectrometry; ^1^H-NMR: Hydrogen-1 nuclear magnetic resonance spectrometry; COSY: (Homonuclear chemical shift) correlation spectroscopy; UPLC-MS: Ultra performance liquid chromatography-mass spectrometry; GC-MS: Gas chromatography-mass spectrometry; MS: Mass spectrometry; HPLC: High-performance liquid chromatography.

### 2.7. Others

**Figure 8:**
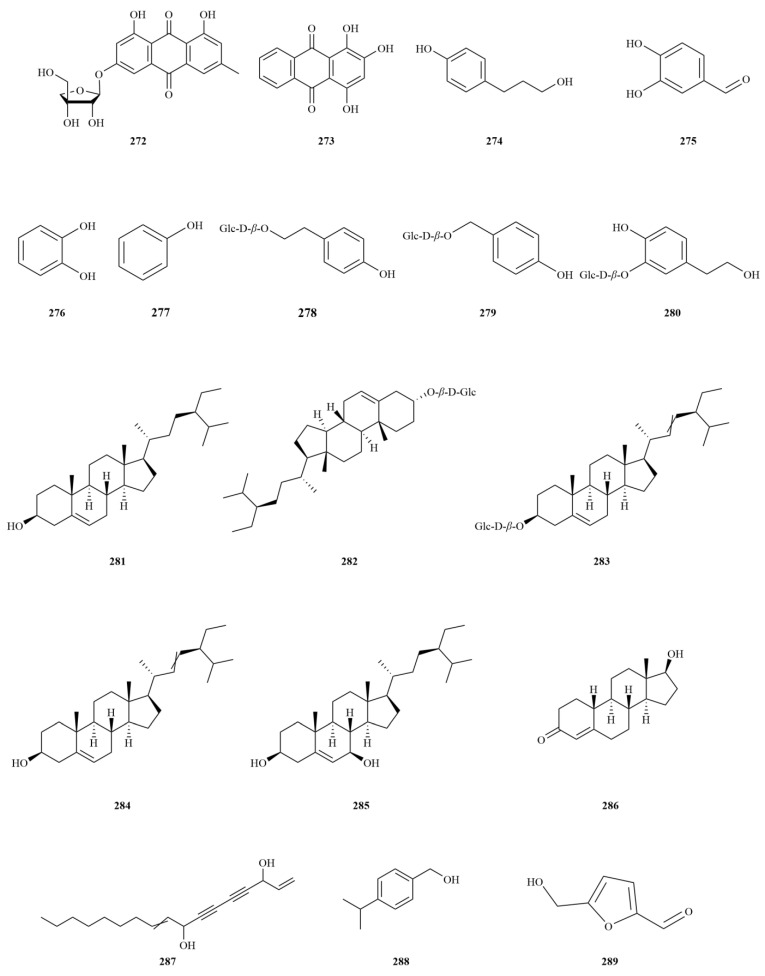

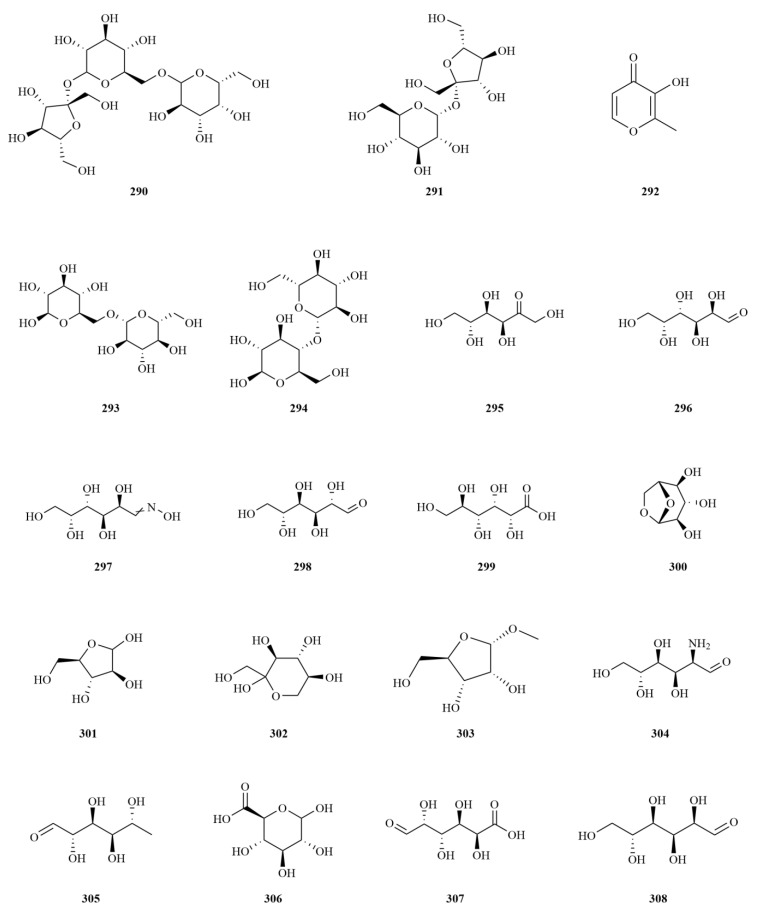

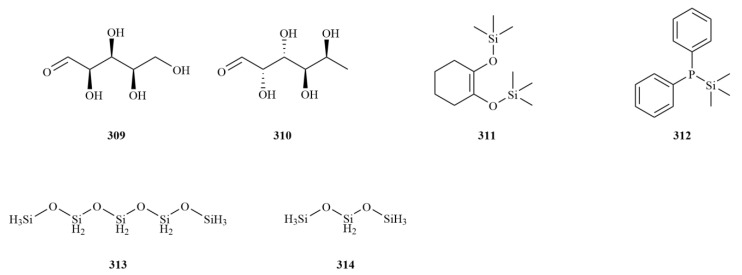
Chemical structures of others isolated from *Eleutherococcus sessiliflorus.* Chemical structures were drawn using Chemdraw Professional 15.0 software.

In addition to the abovementioned compounds, several others have been isolated from *E. sessiliflorus*. Currently, two quinones, frangulin B (**272**) and purpurin (**273**) have been identified. Purpurin shows antigenotoxic, anticancer, neuromodulatory, and antimicrobial potential associated with antioxidant action [77]. Seven phenolics and derivatives (**274**–**280**), six steroids (**281**–**286**), three alcohol compounds (**287**–**289**), twenty-one glycosides (**290**–**310**), and four silicon-containing compounds (**311**–**314**) (Table 7, Figure 8) have also been identified. Most of these compounds are secondary metabolites found in the leaves of *E. sessiliflorus*.

## 3. Pharmacological Activities

In recent years, with the rapid development and widespread recognition of traditional Chinese medicine, pharmacological research focusing on *E. sessiliflorus* has surged [13]. This increased attention from scholars has resulted in significant progress in understanding the pharmacological properties of *E. sessiliflorus*. The therapeutic effects of *E. sessiliflorus* are closely tied to its chemical constituents, which comprise a diverse array of compounds, including terpenoids, flavonoids, and phenylpropanoids. These bioactive compounds contribute to various pharmacological benefits associated with *E. sessiliflorus*, including antioxidative, anti-aging, anti-stress, anti-platelet aggregation, and anti-tumor effects.

### 3.1. Antioxidant Activity

Antioxidants are known for their ability to reduce oxidative stress, slow down oxidation processes, and preserve food quality while preventing degenerative diseases. *E. sessiliflorus* exhibits potent antioxidant activity due to the synergistic effect of its chemical constituents [79]. The flavonoids present in *E. sessiliflorus* extract have shown a strong correlation with hydroxyl radical scavenging activity. *E. sessiliflorus* methanol extract has been found to possess the highest capacity for scavenging hydroxyl radicals (82.35 ± 1.54%) compared to other plants, including *Astragalus membranaceus*, *Polygonatum stenophyllum*, and *Angelica gigas* [80].

Moreover, the polyphenols found in *E. sessiliflorus* also exhibit notable antioxidant activity. The extracts derived from the roots, stems, leaves, and fruits of *E. sessiliflorus* have demonstrated significant nitrite-scavenging capacity (76.00–81.50%) at pH 1.2. Therefore, the aqueous extract of *E. sessiliflorus* has shown the ability to inhibit the formation of nitrosamines in food [81]. *E. sessiliflorus* fruit extract (2 mg/mL) has also been found to inhibit the production of TNF-*α* (decrease of 19 ± 6%) and IL-6 (decrease of 24 ± 3%) induced by lipopolysaccharide, as well as suppress COX-2 luciferase activity (decrease of 98 ± 2%) [82]. These antioxidant and anti-inflammatory effects have led to the incorporation of *E. sessiliflorus* as a functional food ingredient in products such as spicy chicken sauce and wine. For instance, in spicy chicken sauce, *E. sessiliflorus* extract is added at a concentration of 2%, resulting in a high total polyphenol content and strong DPPH and ABTS free radical scavenging activity (12.51 ± 0.33% and 8.43 ± 0.29%) along with anti-bacterial activity [83]. Another popular application of *E. sessiliflorus* is incorporating *E. sessiliflorus* seeds into pork meat Wanja. In this case, *E. sessiliflorus* seeds are added at a concentration of 0.5%, along with *Cinnamomum lureitri* at 1.0% and *Angelica gigas* Nakai at 0.5%. This combination significantly reduces the acidity and peroxide values of pork meat Wanja, extending its shelf life by up to ten days [84].

The antioxidant activities of *E. sessiliflorus* can also be harnessed in beverages and wine. The addition of *E. sessiliflorus* juice to fruit juice or *E. sessiliflorus* extract to wine not only enhances the antioxidant activity (scavenging rates of DPPH and ABTS were 64.80% and 73.30%) of the products but also improves their taste by reducing acidity and bitterness [85,86].

### 3.2. Anti-Aging Activity

The antioxidative activity of *E. sessiliflorus* is particularly noteworthy. Free radicals and oxidative stress play a crucial role in the aging process, and *E. sessiliflorus* has shown potential in combating these challenges. Cellular antioxidants, endogenous (such as glutathione and vitamin E) and derived from dietary sources, can scavenge free radicals and alleviate cellular oxidative stress. Recognizing the importance of identifying natural and safe plant sources with potent free radical scavenging capabilities, research has focused on exploring *E. sessiliflorus* extracts. These extracts have exhibited significant improvements in resistance to oxidative stress. For instance, *E. sessiliflorus* extract has been found to enhance the survival rate of *Caenorhabditis elegans* under oxidative stress conditions. The 500 mg/L stem extract has demonstrated a notable improvement in the thermotolerance of *Caenorhabditis elegans*, increasing their survival time after exposure to ultraviolet radiation by 13.3% [87]. 

Similarly, the leaf extract (500 mg/L) has been shown to enhance thermotolerance, increasing the survival of *Caenorhabditis elegans* up to 57.2 ± 5.30% without affecting their reproductive capacity [88]. Notably, the root extract has augmented the antioxidant capacity of mice, increasing their survival time after heat shock (77.7 ± 8.87%) and ultraviolet irradiation (31.1%). Additionally, the 500 mg/L root extract has shown a protective effect against human A*β* amyloid-induced toxicity in *Caenorhabditis elegans* (*p* < 0.001), suggesting a potential role in regulating the aging process in these nematodes [89]. It is worth noting that the effects of *E. sessiliflorus* extracts may vary among different plant parts. For instance, various parts’ extracts show varying resistances to ultraviolet radiation, with only the stems’ extract significantly reducing DNA oxidative damage in rat lymphocytes. Therefore, it is essential to study the distinct parts of *E. sessiliflorus* separately to further explore its medicinal and nutritional applications in the future.

Furthermore, investigations have been carried out to uncover the anti-aging effects of *E. sessiliflorus* using *Drosophila melanogaster* as a model organism. Results have indicated that the ethyl acetate extract fraction and *n*-butanol extract fraction of the alcohol extract from *E. sessiliflorus* leaves exhibited significant extensions in the lifespan of *Drosophila melanogaster* (8.20–21.43%) within the concentration range of 0.25 mg/mL to 2.5 mg/mL. Interestingly, a concentration-dependent trend was observed, where the extension rate initially increased with the rise in extract concentration. Drosophila *melanogaster* had the longest lifespan at 1.25 mg/mL, subsequently decreasing. The above findings provide additional evidence supporting the potential anti-aging properties of *E. sessiliflorus* [90]. However, the precise underlying mechanism of action behind these effects is yet to be fully elucidated.

### 3.3. Anti-Stress Activity

The fruits of *E. sessiliflorus* contain polymeric and glycosidic compounds composed of a series of monosaccharides, including mannose, rhamnose, and glucose. These compounds have demonstrated significant anti-stress effects, such as anti-fatigue properties, tolerance to hypoxia, and the enhancement of immune regulatory capacity. Notably, these effects have been observed at two different dosage levels of the fruit polysaccharides (200 mg/kg and 400 mg/kg), highlighting their pronounced anti-fatigue properties. Specifically, mice administered the 400 mg/kg dose showed a doubling of swimming time compared to the control group, indicating improved carbon particle clearance ability with enhanced swallow index (0.0613 ± 0.0067) and swallow coefficient (6.559 ± 0.518) in mice. Furthermore, a dose of 200 mg/kg significantly improved the survival time of mice under hypoxia (32.48 ± 2.99 min) [78,91].

Furthermore, the polyphenols present in *E. sessiliflorus* (100 mg/kg and 200 mg/kg) have been shown to significantly prolong the exhaustive swimming time of mice by 14.35 and 17.38 min, respectively. These polyphenols inhibit the depletion of liver glycogen, raising its levels from 2.53 mg/mL in the control group to 2.92 mg/mL and 3.03 mg/mL. Moreover, they increase muscle glycogen content by 28.82% and 35.08%, enhance the activity of glutathione peroxidase in mice (114.67 U/mL and 109.62 U/mL), and reduce lactate levels by 10.96% and 17.44% and creatine kinase levels by 26.43 ng/mL and 26.57 ng/mL. Collectively, these effects contribute to elevated resistance to fatigue in mice [92].

*E. sessiliflorus* also exhibits sedative and hypnotic effects. The combination of *E. sessiliflorus* fruit extract (1175 mg/kg and 585 mg/kg) with barbiturate drugs (60 mg/kg) synergistically prolongs the sleep time induced by pentobarbital sodium. In animals administered sub-threshold hypnotic doses (30 mg/kg) of pentobarbital sodium, *E. sessiliflorus* induces a sleep state. Additionally, *E. sessiliflorus* fruit extract (810 mg/kg) reduces animal locomotion and impairs motor coordination [93].

Similarly, the combined administration of the aqueous and alcohol extracts of *E. sessiliflorus* leaves and fruits with 5-hydroxytryptophan has been shown to induce sleep in mice. Notably, the ethanolic extract of the fruits (32 mg/kg) significantly increased the mice’s sleep onset rate. Additionally, the aqueous and alcoholic extracts of fruits or leaves (8 mg/kg) exhibited a synergistic effect with 5-hydroxytryptophan (2.5 mg/kg). This combination also alleviated insomnia induced by *p*-chlorophenoxyacetic acid. These extracts counteracted the stimulatory effects caused by flumazenil and thiosemicarbazide in mice, suggesting a correlation between their sedative effects and the neurotransmitter systems involving 5-hydroxytryptamine and *γ*-aminobutyric acid.

Moreover, the alcohol extract of *E. sessiliflorus* leaves and fruits exhibited stronger sedative and hypnotic activities than the aqueous extract. This difference can be attributed to higher levels of isofraxidin, a component known for its sedative and hypnotic effects, in the alcohol extract. Saponin components also significantly contributed to the sedative and hypnotic effects [94,95].

Network pharmacology analysis has identified the targets underlying the sedative and hypnotic effects of *E. sessiliflorus*. Enzymes constitute the highest proportion of these targets (17.44%), followed by receptors (25.00%), including 5-hydroxytryptamine and *γ*-aminobutyric acid-A receptors. This finding suggests that the sedative and hypnotic effects of *E. sessiliflorus* primarily involve modulating specific enzymes and receptors [20].

### 3.4. Anti-Platelet Aggregation Activity

Platelet aggregation, which occurs when platelets are exposed to external stimuli, is a crucial step in the formation of platelet thrombi, leading to thrombotic diseases. These diseases pose a significant threat to human health, with high mortality and disability rates. While various medications have been developed to treat thrombotic conditions, their adverse reactions, such as bleeding tendencies, gastrointestinal discomfort, and hepatotoxicity, have propelled researchers to focus on developing naturally safe therapeutic agents.

Studies have investigated the effects of water decoctions and active components (total saponins, total flavonoids, and lupane-type triterpenoid saponins) derived from the leaves of *E. senticosus* and *E. sessiliflorus* (concentrations of both 25 mg/kg and 100 mg/kg) on adenosine diphosphate-induced anti-platelet aggregation and antithrombotic activity. These investigations assessed toxicity-related parameters, including mouse platelet toxicity, prothrombin time, bleeding time, mouse tail length, and the occurrence rate of tail necrosis. The results revealed that the flavonoids in both *E. senticosus* and *E. sessiliflorus* leaves exhibited varying degrees of inhibition of thrombus formation induced by carrageenan in mice. This inhibition led to a reduction in the length of thrombus formation in the mouse tail. Moreover, the flavonoids (25 mg/kg and 100 mg/kg) increased the levels of serum cAMP (3.10 ± 0.22 nM and 3.19 ± 0.31 nM) in mice and suppressed the abnormally elevated serum TXB2 induced (1.28 ± 0.20 nM and 0.95 ± 0.12 nM) by carrageenan, with the most prominent effects observed with the total flavonoids from *E. sessiliflorus* leaves [96].

Furthermore, *E. sessiliflorus* fruits contain a significant amount of oleanolic acid, which has demonstrated a notable anti-platelet aggregation effect [18]. Additionally, three novel triterpenoids isolated from the fruits have exhibited similar anti-platelet aggregation effects to acetylsalicylic acid in vitro experiments. Administration of a 70% ethanol extract of *E. sessiliflorus* fruits at a dose of 1000 mg/kg in rats resulted in the highest inhibition of platelet aggregation, reaching 61.3%. Both in vitro and in vivo studies have confirmed the significant anti-platelet aggregation and antithrombotic effects of these compounds. Furthermore, they have been shown to influence the release of adenosine diphosphate, thus impacting platelet aggregation [97].

The above findings highlight the potential of *E. sessiliflorus*, particularly its flavonoids and triterpenoids, as effective natural agents for inhibiting platelet aggregation and preventing thrombotic diseases. Further research and exploration of the underlying mechanisms behind these effects are warranted to fully understand the therapeutic potential of *E. sessiliflorus* in the context of thrombotic conditions.

### 3.5. Effects on Glucose Metabolism

*E. sessiliflorus* roots (5 mg/kg) have shown promising effects in accelerating the decline in alimentary hyperglycemia concentration and increasing hepatic glycogen content [98]. Moreover, *E. sessiliflorus* leaves have demonstrated the ability to significantly lower blood glucose levels in diabetic rats, improve lipid metabolism, reduce total cholesterol and low-density lipoprotein levels, and elevate high-density lipoprotein levels. Notably, the concentration of triglycerides decreased by 60.80% compared to the control group [99]. Additionally, an herbal formulation containing *E. sessiliflorus*, along with *Panax ginseng*, *Astragalus membranaceus*, *Glycyrrhiza uralensis*, and other herbs, has exhibited remarkable improvements in blood glucose (442.50 ± 36.00 mg/dL), cholesterol (159.20 ± 18.40 mg/dL), blood glycated hemoglobin (6.30 ± 0.8 mg/dL), and plasma triglyceride levels (99.40 ± 15.00 mg/dL) in db/db mice (C57BL/Ks mice), which are commonly used as a model for diabetes. This formulation holds potential for the prevention and treatment of diabetes and its complications [100]. Further research has shown that the stems of *E. sessiliflorus* affect diabetes and its complications by inhibiting the activity of aldose reductase [101]. Moreover, the flavonoids present in the stems of *E. sessiliflorus* (100 mg/kg and 300 mg/kg) can regulate the upregulation of INSRR, HNF1A, and GLUT10 expression, thereby modulating blood glucose levels and alleviating disruptions in lipid metabolism [102].

The above findings emphasize the potential of *E. sessiliflorus* as a natural remedy for managing glucose metabolism and addressing issues associated with diabetes. However, further research is necessary to explore the underlying mechanisms and optimize the utilization of *E. sessiliflorus* in diabetes treatment.

### 3.6. Effects on the Cardiovascular System

The polyphenols extracted from *E. sessiliflorus* fruits (75 mg/kg and 150 mg/kg) have been found to significantly reduce the protein expression levels of intercellular cell adhesion molecule-1, vascular cell adhesion molecule-1, phospho-p38, and phospho-ERK1/2. As a result, they can effectively lower serum lipid levels, adhesion molecule levels, and inflammatory factor levels in rats, reducing lipid deposition in the aorta. This demonstrates a preventive effect against atherosclerosis [103].

Furthermore, the 3,4-seco-lupane triterpenoids (chiisanoside, divaroside, sessiloside-A, and chiisanogenin) found in *E. sessiliflorus* leaves exhibit potent antiarrhythmic activity. They are capable of reducing malondialdehyde levels, increasing serum superoxide dismutase levels, and maintaining the expression levels of Na^+^-K^+^-ATPase, Ca^2+^-Mg^2+^-ATPase, and apoptosis-related proteins. Among these, divaroside (41.6 mg/kg) has shown superior efficacy in treating ventricular arrhythmias induced by BaCl_2_. The unique glycosyl chain structure of divaroside may contribute to its effectiveness in sustaining the expression of PKA, thus improving its antiarrhythmic properties [104]. Other terpenoids found in *E. sessiliflorus* have also demonstrated potent inhibitory activity against ACE, ranging from 1.8 μg/mL to 2.9 μg/mL, enhancing blood flow and exerting antihypertensive effects [8].

Moreover, the ethanol extract of *E. sessiliflorus* fruits (2, 20, and 200 μg/mL) has shown the ability to enhance the expression of endothelial nitric oxide (NO) synthase, thereby increasing the production of endothelial NO. This was assessed by measuring the fluorescence intensity of 4-amino-5-methylamino-2′,7′-difluoro-luorescein diacetate (with the control group set at 100%), which showed values of 127.54 ± 14.10%, 141.47 ± 8.16%, and 167.54 ± 8.41%, respectively. This enhanced endothelium-dependent NO release is known to improve vasodilation and blood circulation, providing significant benefits to the cardiovascular system. The vasorelaxation capability of *E. sessiliflorus* fruits is similar to that of *Ginkgo biloba* leaf extract, further contributing to its potential cardiovascular benefits. Additionally, the ethanol extract of *E. sessiliflorus* fruits is similar to captopril, a commonly used ACE inhibitor, in significantly reducing ACE activity and improving vasodilation in spontaneously hypertensive rats, thereby lowering blood pressure. The hypotensive effect of a high-dose ethanol extract of *E. sessiliflorus* fruits (600 mg/kg) is comparable to that of captopril (100 mg/kg) [11,105].

During metabolomic investigations of *E. sessiliflorus* fruits, significant variations in the metabolic process have been observed. The hypotensive effect of *E. sessiliflorus* fruits is achieved through the amelioration of negative metabolic effects associated with hypertension. This mechanism differs slightly from the metabolic process induced by captopril, a commonly used hypotensive medication. Notably, the presence of succinate and betaine within the metabolic products of *E. sessiliflorus* fruits suggests their potential utility as biomarkers for its hypotensive effect [106].

In addition to its hypotensive effects, *E. sessiliflorus* demonstrates remarkable potential in ameliorating various parameters associated with metabolic health. Animal studies have revealed that the ethanol extract of *E. sessiliflorus* roots (500 mg/kg and 700 mg/kg) can effectively mitigate adverse changes induced by a high-fat diet. Specifically, *E. sessiliflorus* has been found to improve body weight management by preventing excessive weight gain. After administering 500 mg/kg to mice, their body weight decreased to 84.00 ± 7.00% (compared to the blank group) [107]. Moreover, the aqueous-alcohol extract of *E. sessiliflorus* fruits (3 mg) significantly influences lipid metabolism, as evidenced by its ability to reduce total cholesterol, triglyceride, and free fatty acid levels. This comprehensive modulation of lipid profiles contributes to the potential therapeutic role of *E. sessiliflorus* in combating hyperlipidemia [108]. The above findings highlight the diverse metabolic effects of *E. sessiliflorus* fruits, contributing to their potential therapeutic applications in managing hypertension, hyperlipidemia, and related cardiovascular conditions. 

### 3.7. Effects on the Immune System

Polysaccharides are considered vital bioactive components in plants and hold immense research potential and economic value. *E. sessiliflorus* is rich in polysaccharides, which exhibit diverse effects, including scavenging free radicals, combating fatigue, enhancing tolerance to hypoxia, and boosting immune regulatory capabilities. Initial studies on *E. sessiliflorus* have isolated three polysaccharides that demonstrate varying degrees of immune regulatory activity within the concentration range of 25–200 μg/mL. These polysaccharides have shown the ability to stimulate lymphocyte proliferation, enhance phagocytosis in peritoneal macrophages, elevate NO release, and activate the cytokine TNF-*α* [109]. This comprehensive array of activities highlights their immunomodulatory effects, underscoring their vast prospects and economic significance.

In addition, *E. sessiliflorus* extracts have been found to positively affect the immune system in both normal and tumor-bearing mice. In normal animals, the roots of *E. sessiliflorus* extract (300 mg/kg) enhance the restoration of spleen and thymus weights after forced swimming experiments while increasing spleen cell counts and immune-related cytokine TNF-*α* levels. However, their impact on the expression of IFN-*γ* and IL-2 is comparatively less pronounced [110]. In tumor-bearing mice, the ethanol extract of *E. sessiliflorus* roots (500 mg/kg) demonstrates anticancer effects by promoting immune cell proliferation and enhancing macrophage NO production without adversely affecting the proliferation of normal mouse spleen cells [111].

The immunomodulatory effects of *E. sessiliflorus* are not limited to the pharmaceutical industry but also play a significant role in enhancing disease resistance in livestock. The ethanol extract of *E. sessionliflorus* fruits has been demonstrated to enhance the vitality and growth rate of 3D4/31 porcine macrophages in a concentration-dependent manner. At 120 μg/mL, it maximally increased cell viability by 11.73% ± 2.02% while upregulating intracellular reactive oxygen species (ROS) levels. Furthermore, pretreatment with the fruits’ ethanol extract augments the in vitro bactericidal activity of these cells against *Escherichia coli*. Intriguingly, the fruits’ ethanol extract, similar to phorbol 12-myristate 13-acetate, effectively improves the expression levels of NF-*κ*B and TNF-*α*, which in turn influence lipid synthesis and fatty acid oxidation metabolism.

Moreover, the combination of both the fruits’ ethanol extract (120 μg/mL) and phorbol 12-myristate 13-acetate demonstrates therapeutic potential in restoring NF-*κ*B, TNF-*α*, and lipid metabolism levels. This combination holds promise as an effective feed additive to enhance the immunity of livestock [112]. The above findings highlight the multifaceted immunomodulatory effects of *E. sessiliflorus*, not only in human health but also in the agricultural sector, showcasing its potential for commercial applications in medicine and livestock feed supplements.

### 3.8. Anti-Tumor Activity

*E. sessiliflorus* has been found to possess significant anti-tumor activity, primarily attributed to its diverse array of bioactive compounds. Triterpenoids isolated from *E. sessiliflorus* have shown promising anti-tumor effects. For instance, chiisanoside, derived from *E. sessiliflorus*, exhibits in vivo anti-tumor activity by promoting cell apoptosis and inhibiting angiogenesis. In mice bearing the H22 tumor, chiisanoside (120 mg/kg and 240 mg/kg) effectively suppresses tumor growth while upregulating the expression of cytokines such as IL-2, TNF-*α*, and IFN-*γ*. Furthermore, it demonstrates rapid absorption in vivo and shows targeting properties towards the liver and small intestine [113].

Another triterpenoid with anti-tumor potential in *E. sessiliflorus* fruits is calenduloside E 6′-methyl ester, an oleanane-type compound. This triterpenoid has been reported to induce apoptosis in CT-26 mouse colon cancer cells in the range of 2.5 to 25 µM. The number of cells in the sub-G1 population increased from 5.1% to 99.1%, respectively. Furthermore, it can inhibit tumor growth in the CT-26 animal model. The induction of apoptosis by calenduloside E 6′-methyl ester is mediated by activating the caspase cascade, which plays a crucial role in apoptotic mechanisms [34]. Similarly, sessiligenin, another compound found in *E. sessiliflorus*, exerts its effects by modulating multiple targets within the PI3K/AKT signaling pathway, ultimately leading to apoptosis induction in HepG2 cells [114].

*E. sessiliflorus* also contains other compounds that exhibit significant anti-tumor activities. Hyperoside, for example, stands out for its ability to inhibit ERK activity, which suppresses the transactivation of activator protein 1 and the phosphorylation of p90RSK, CREB, and STAT3 induced by ultraviolet radiation. Activator protein 1 is a key transcription factor involved in inflammation and various cancers, including skin, breast, and cervical [115]. Cyanidin-3-O-sambubioside, another compound in *E. sessiliflorus*, has been found to effectively reduce the secretion and expression of matrix metalloproteinase-9 within the concentration range of 1–30 μg/mL, thereby suppressing the metastatic process of breast cancer cells, particularly in aspects related to angiogenesis and invasion [12].

Furthermore, the stem bark extract of *E. sessiliflorus* (50 μg/mL) has demonstrated the ability to inhibit tumor growth and modulate immune activity through various pathways. On the one hand, it induces non-apoptotic cell death in human breast cancer cells (MDA-MB-231 and MCF-7) through ROS-dependent and ROS-independent mechanisms involving mitochondrial induction [116]. On the other hand, it (1 μg/mL and 10 μg/mL) exerts tumor-inhibitory effects by promoting NO production by macrophages, accelerating thymocyte proliferation, and inhibiting tumor cell proliferation [117]. The above findings highlight *E. sessiliflorus* as a valuable medicinal herb with diverse anti-tumor constituents, positioning it as a significant asset in anti-tumor therapies.

### 3.9. Other Pharmacological Effects

*E. sessiliflorus* possesses a wide range of pharmacological effects recognized in recent studies. One notable effect is its potential as an analgesic agent. *E. sessiliflorus* has shown the ability to ameliorate formalin-induced pain, making it suitable for relieving both general and neuropathic pain from nerve injury [118]. Triterpenoids and polyphenols in *E. sessiliflorus* have inhibited BV2 and RAW264.7 cells induced by lipopolysaccharides. These compounds effectively reduce the production of inflammatory mediators such as NO, PGE2, TNF-*α*, IL-1*β*, and IL-6, thereby exerting potent anti-inflammatory effects [9,16,83]. Additionally, the root barks of *E. sessiliflorus* have been found to inhibit osteoclast differentiation activated by RANKL in bone marrow macrophages and prevent bone loss induced by ovariectomy [119]. Moreover, *E. sessiliflorus* has demonstrated chondrogenic regulatory activity by enhancing the mRNA expression of markers associated with cartilage formation [120]. The above findings highlight the therapeutic potential of *E. sessiliflorus* in treating various bone-related disorders. Furthermore, *E. sessiliflorus* possesses neuroprotective effects [121], hepatoprotective effects [122], and protective effects on the gastrointestinal tract [123]. These additional pharmacological effects further broaden the potential applications of *E. sessiliflorus* in various health conditions.

## 4. Discussion

We have provided a comprehensive overview of the phytochemistry and biological activities of *E. sessiliflorus*, a traditional medicinal plant. With a total of 314 reported compounds, *E. sessiliflorus* exhibits a diverse range of phytochemicals, including triterpenoids, monoterpenoids, sesquiterpenoids, simple phenylpropanoids, lignans, coumarins, flavonoids, volatile oils, organic acids (esters), nitrogenous compounds, quinones, phenolics, and carbohydrates. We have also elucidated the various biological activities of these compounds and *E. sessiliflorus* extracts. An extensive review of the literature shows that *E. sessiliflorus* possesses antioxidant, anti-aging, anti-stress, antiplatelet aggregation, and anticancer effects. Additionally, it shows improvements in glucose metabolism, cardiovascular health, and immune system function. Triterpenoids and lignans, in particular, are identified as the primary constituents responsible for mediating these biological activities.

In addition, the root barks of *E. sessiliflorus* have long been renowned for their medicinal properties, prominently mentioned in the “Chinese Materia Medica” alongside *Eleutherococcus nodiflorus* as sources of Wujia Pi. Consequently, extensive scholarly research has been dedicated to unraveling the chemical composition of *E. sessiliflorus* root barks. In recent years, the approval of *E. sessiliflorus* as a new resource food has fueled a surge in demand from both domestic and international markets. As a result, large-scale cultivation of *E. sessiliflorus* has been initiated, inspiring researchers to explore different medicinal parts of the plant and broaden the scope of their investigations. Among these parts, the fruits of *E. sessiliflorus* have gained attention due to their distinct characteristics. With their blackberry-like appearance, they exhibit a diverse range of biological activities while maintaining a high safety profile. The fruits possess favorable taste qualities, making them an emerging resource for research and processing in the food industry. However, current studies on the chemical composition of *E. sessiliflorus* primarily focus on the isolation and identification of individual compounds, with limited research on variations in component content among different regions and plant parts. Moreover, the establishment of quality standards for *E. sessiliflorus* remains an area that requires further exploration. Emphasizing future research in these aspects is crucial to enhance the standardized application and quality control of *E. sessiliflorus*.

Of particular interest within *E. sessiliflorus* are the 3,4-seco-lupane triterpenoids, a unique class of compounds exclusively found in the *Eleutherococcus* genus. To date, researchers have discovered thirty-six compounds belonging to this class in *E. sessiliflorus*. These compounds exhibit variations in their chemical structures, such as hydroxyl substitutions, deoxidization, and glycoside linkage positions, while the basic structure revolves around chiisanoside. Notably, specific compounds found only in *E. sessiliflorus*, such as elesesterpene A-K and acanthosessilioside A-O, have been identified, adding to the distinct chemical profile of *E. sessiliflorus*. Exploring the biological activities of these 3,4-seco-lupane triterpenoids presents an exciting avenue for further in-depth investigations, potentially uncovering safe and effective compounds with potential therapeutic applications.

Furthermore, extensive research has revealed a wide range of pharmacological effects that *E. sessiliflorus* possesses. These effects mainly focus on its anti-tumor properties and ability to protect the cardiovascular system. However, it is worth noting that some studies have only used extracts of *E. sessiliflorus* instead of pure compounds. Furthermore, investigations into other biological activities of *E. sessiliflorus* are not sufficiently comprehensive, and many underlying mechanisms have not been elucidated. Therefore, there is a need for further in-depth exploration of the pharmacological activities of *E. sessiliflorus*.

Also, the tender leaves of *E. sessiliflorus* are popular as a wild vegetable and can be dried to make tea. This tea is known for its ability to replenish qi, strengthen the spleen, and calm the mind. The fruits of *E. sessiliflorus* are used in beverages and wines, while the seeds are employed as spice. Building upon the known pharmacological effects of *E. sessiliflorus*, its applications extend to pharmaceuticals for anti-inflammatory and antioxidant purposes and its use in feed additives with antimicrobial properties. Additionally, *E. sessiliflorus* serves as a food additive in beverages and wines. Given its notable safety profile and high pharmacological effect, *E. sessiliflorus* warrants an in-depth investigation of its various applications. It is essential to closely monitor the in vivo metabolic patterns of its bioactive constituents while ensuring the maintenance of therapeutic efficacy. Current research on the pharmacokinetics of *E. sessiliflorus* is limited, despite its significance in unraveling metabolic pathways. These investigations contribute to understanding its mechanisms of action but also aid in identifying quality markers for *E. sessiliflorus*.

Indeed, this review has certain limitations. Firstly, the methods used to collect literature and data were subject to limitations, which may have resulted in the exclusion of certain relevant studies. Additionally, the quality of research reports was not effectively evaluated, which may have impacted the reliability of the findings in this review. Another notable limitation is the lack of coverage of studies related to toxicity and clinical relevance. There is a scarcity of relevant reports, and the few toxicity research reports that do exist are relatively preliminary. Furthermore, there are no reports of clinical research on extracts or compounds from *E. sessiliflorus*. Moving forward, future researchers should aim to address these limitations and explore the uncharted territory of toxicity and clinical relevance.

## 5. Conclusions

*E. sessiliflorus*, a medicinal and edible plant belonging to the same botanical family as *Panax ginseng* and displaying chemical constituents similar to *E. senticosus*, presents promising prospects for extensive research. However, no comprehensive and detailed report is available on its components and pharmacological effects. Therefore, the primary objective of this review is to comprehensively elucidate the research conducted on *E. sessiliflorus* from two perspectives, phytochemistry and pharmacological effects, by conducting searches across multiple databases. The components are classified into seven categories, and their pharmacological effects are categorized into nine classifications for summary and discussion. This review aims to establish a strong foundation for further exploration into the potential uses of *E. sessiliflorus* and guides future research endeavours in this field.

## Figures and Tables

**Table 4:** Volatile oils isolated from *Eleutherococcus sessiliflorus.*

NO.	Name	Formula	Exact Theoretical Molecular Weight	Source	Characterization Method	Refs.
**165**	Hexanoic acid	C_6_H_12_O_2_	116.0837	roots	GC	[66]
**166**	Octanoic acid	C_8_H_16_O_2_	144.1150	roots	GC	[66]
**167**	Methyl salicylate	C_8_H_8_O_3_	152.0473	roots	GC	[66]
**168**	Aromadendrene	C_15_H_24_	204.1878	roots	GC	[66]
**169**	Nerolidol	C_15_H_26_O	222.1984	roots, stem barks	GC-MS, GC	[64,66]
**170**	Ledol	C_15_H_26_O	222.1984	roots	GC	[66]
**171**	(−)-Spathulenol	C_15_H_24_O	220.1827	roots, stem barks	GC-MS, GC	[64,66]
**172**	Levomenol	C_15_H_26_O	222.1984	roots, stem barks	GC-MS, GC	[64,66]
**173**	Tetradecanal	C_14_H_28_O	212.2140	roots	GC	[66]
**174**	Espatulenol	C_15_H_24_O	220.1827	roots	GC	[66]
**175**	Heptadecane	C_17_H_36_	240.2817	roots	GC	[66]
**176**	(E,E)-Farnesol	C_15_H_26_O	222.1984	roots, stem barks	GC-MS, GC	[64,66]
**177**	Farnesal	C_15_H_24_O	220.1827	roots, stem barks	GC-MS, GC	[64,66]
**178**	Diisobutyl phthalate	C_16_H_22_O_4_	278.1518	roots	GC	[66]
**179**	Methyl hexadecanoate	C_17_H_34_O_2_	270.2559	roots	GC	[66]
**180**	Octadecane	C_18_H_38_	254.2974	roots	GC	[66]
**181**	(R)-(−)-Falcarinol	C_17_H_24_O	244.1827	roots	GC	[66]
**182**	Tricosane	C_23_H_48_	324.3756	roots	GC	[66]
**183**	Octanal	C_8_H_16_O	128.1201	stem barks	GC-MS	[64]
**184**	4-Isopropenyl-1-methylcyclohexene	C_10_H_16_	136.1252	stem barks	GC-MS	[64]
**185**	(R)-3,7-Dimethyloct-6-en-1-ol	C_10_H_20_O	156.1514	stem barks	GC-MS	[64]
**186**	(E)-2-Decenal	C_10_H_18_O	154.1358	stem barks	GC-MS	[64]
**187**	(1S-Endo)-1,7,7-trimethyl-bicyclo [2.2.1]heptan-2-ol-acetate	C_12_H_20_O_2_	196.1463	stem barks	GC-MS	[64]
**188**	(E,E)-2,4-Decadienal	C_10_H_16_O	152.1201	stem barks	GC-MS	[64]
**189**	2,6-Dimethyl-2,6-octadiene	C_10_H_18_	138.1409	stem barks	GC-MS	[64]
**190**	(E)-3,7-Dimethyl-2,6-octadien-1-ol acetate	C_12_H_20_O_2_	196.1463	stem barks	GC-MS	[64]
**191**	(−)-Trans-myrtanyl acatate	C_12_H_20_O_2_	196.1463	stem barks	GC-MS	[64]
**192**	Geranylacetone	C_13_H_22_O	194.1671	stem barks	GC-MS	[64]
**193**	1-(1,5-Dimethyl-4-hexenyl)-4-methylbenzene	C_15_H_22_	202.1722	stem barks	GC-MS	[64]
**194**	(E,E)-3,7,11-Trimethyl-1,3,6,10-dodecatetraene	C_15_H_24_	204.1878	stem barks	GC-MS	[64]
**195**	S-1-Methyl-4-(5-methyl-1-methylene-4-hexenyl)cyclohexene	C_15_H_24_	204.1878	stem barks	GC-MS	[64]
**196**	[1R-(1*α*,3a*β*,4*α*,7*β*)]-1,2,3,3a,4,5,6,7-Octahydro-7-isopropenyl-1,4-dimethylazulen	C_15_H_24_	204.1878	stem barks	GC-MS	[64]
**197**	E,E-3,7,11,15-Tetramethyl-1,6,10,14-hexadecatetraen-3-ol	C_20_H_34_O	290.2610	stem barks	GC-MS	[64]
**198**	(E,E)-3,7,11-Trimethyl-2,6,10-dodecatrien-1-ol acetate	C_17_H_28_O_2_	264.2089	stem barks	GC-MS	[64]
**199**	Hentriacontane	C_31_H_64_	436.5008	stem barks	GC-MS	[64]
**200**	Hexatriacontane	C_36_H_74_	506.5791	stem barks	GC-MS	[64]
**201**	Octacosane	C_28_H_58_	394.4539	stem barks	GC-MS	[64]
**202**	9-Octyl-heptadecane	C_25_H_52_	352.4069	stem barks	GC-MS	[64]
**203**	8-Heptyl-pentadecane	C_22_H_46_	310.3600	stem barks	GC-MS	[64]
**204**	Borneol	C_10_H_18_O	154.1358	stems	GC-MS	[65]
**205**	Butylated hydroxytoluene	C_15_H_24_O	220.1827	stems	GC-MS	[65]
**206**	Nonadecane	C_19_H_40_	268.3130	stems	GC-MS	[65]
**207**	Dibutyl phthalate	C_16_H_22_O_4_	278.1518	stems	GC-MS	[65]
**208**	*α*-Bergamotene	C_15_H_24_	204.1878	roots	GC	[66]
**209**	*β*-Caryophyllene oxide	C_15_H_24_O	220.1827	roots	GC	[66]
**210**	*α*-Gurjunene	C_15_H_24_	204.1878	roots	GC	[66]
**211**	9,17-Diene-octodecane	C_18_H_34_	250.2661	roots	GC	[66]
**212**	5-Methyl-2-(1-methylethyl)-cyclohexanol	C_10_H_20_O	156.1514	roots	GC	[66]
**213**	*α*-Neoclovene	C_15_H_24_	204.1878	roots	GC	[66]
**214**	3,7,11,15-Tetramethyl-2,6,10,14-hexadecatetraene-1-ol	C_20_H_34_O	290.2610	roots	GC	[66]
**215**	1,5,5-Trimethyl-6-(2-butenyl)-1-cyclohexene	C_13_H_22_	178.1722	stems	GC-MS	[65]
**216**	*β*-Cedren-9-*α*-ol	C_15_H_24_O	220.1827	stems	GC-MS	[65]
**217**	[1aR-(1*α*a, 4*α*a, 7a, 7a*β*, 7b*α*)]-1a,2,3,5,6,7,7a,7b-Octahydro-1,1,4,7-tetramethyl-1H-cycloprop[e]azulene	C_15_H_24_	204.1878	stem barks	GC-MS	[64]
**218**	Undecane	C_11_H_24_	156.1878	leaves	GC-MS	[67]
**219**	Octane	C_8_H_18_	114.1409	leaves	GC-MS	[67]

GC: Gas chromatography; GC-MS: Gas chromatography-mass spectrometry.

**Table 5:** Organic acids (esters) isolated from *Eleutherococcus sessiliflorus.*

NO.	Name	Formula	Exact Theoretical Molecular Weight	Source	Characterization method	Refs.
**220**	Protocatechuic acid	C_7_H_6_O_4_	154.0266	fruits, root barks	IR, ^13^C-NMR, ^1^H-NMR	[22,53]
**221**	Benzoic acid	C_7_H_6_O_2_	122.0368	fruits	^13^C-NMR, ^1^H-NMR, MS	[48]
**222**	Gallic acid	C_7_H_6_O_5_	170.0215	stems	IR, ^13^C-NMR, ^1^H-NMR	[54]
**223**	Protocatechuicacid methyl ester	C_8_H_8_O_4_	168.0423	fruits	HPLC-MS, ^13^C-NMR, ^1^H-NMR	[30]
**224**	Niduloic acid	C_15_H_16_O_5_	276.0998	fruits	HPLC-MS, ^13^C-NMR, ^1^H-NMR	[30]
**225**	Linoleic acid	C_18_H_32_O_2_	280.2402	fruits, leaves, stem barks	UPLC-MS, IR, ^13^C-NMR, ^1^H-NMR GC-MS	[20,64,69]
**226**	Quinic acid	C_7_H_12_O_6_	192.0634	leaves	UPLC-MS	[20]
**227**	Azelaic acid	C_9_H_16_O_4_	188.1049	leaves	UPLC-MS	[20]
**228**	Malyngic acid	C_18_H_32_O_5_	328.2250	leaves	UPLC-MS	[20]
**229**	Stearidonic acid	C_18_H_28_O_2_	276.2089	leaves	UPLC-MS	[20]
**230**	Parinaric acid	C_18_H_28_O_2_	276.2089	leaves	UPLC-MS	[20]
**231**	Eleostearic acid	C_18_H_30_O_2_	278.2246	leaves	UPLC-MS	[20]
**232**	Pinolenic acid	C_18_H_30_O_2_	278.2246	leaves	UPLC-MS	[20]
**233**	Palmitoleic acid	C_16_H_30_O_2_	254.2246	leaves	UPLC-MS	[20]
**234**	1-Monopalmitin	C_19_H_38_O_4_	330.2770	leaves	GC-MS	[67]
**235**	*o*-Toluic acid	C_8_H_8_O_2_	136.0524	leaves	GC-MS	[67]
**236**	Lactic acid	C_3_H_6_O_3_	90.0317	leaves	GC-MS	[67]
**237**	Butenedioic acid	C_4_H_4_O_4_	116.0110	leaves	GC-MS	[67]
**238**	D-Gluconic acid	C_6_H_12_O_7_	196.0583	leaves	GC-MS	[67]
**239**	Isophthalic acid	C_8_H_6_O_4_	166.0266	leaves	GC-MS	[67]
**240**	Palmitic acid	C_16_H_32_O_2_	256.2402	leaves, stem barks	GC-MS	[64,67]
**241**	Stearic acid	C_18_H_36_O_2_	284.2715	leaves	GC-MS	[67]
**242**	Glycerol monostearate	C_21_H_42_O_4_	358.3083	leaves	GC-MS	[67]
**243**	Erythrono-1,4-lactone	C_4_H_6_O_4_	118.0266	leaves	GC-MS	[67]
**244**	Vanillic acid-4-O-*β*-D-glucopyside	C_14_H_18_O_9_	330.0951	fruits	MS, ^13^C-NMR, ^1^H-NMR	[14]
**245**	4-O-*β*-D-Glucopyranosyl-3,5-dimethoxygallic acid	C_14_H_18_O_9_	330.0951	fruits	MS, ^13^C-NMR, ^1^H-NMR	[14]
**246**	Vanillate glucoside	C_14_H_18_O_9_	330.0951	fruits	MS, ^13^C-NMR, ^1^H-NMR	[14]
**247**	Syringic acid	C_9_H_10_O_5_	198.0528	roots	HPLC-MS	[29]
**248**	Vanillic acid	C_8_H_8_O_4_	168.0423	roots	HPLC-MS	[29]
**249**	4-Hydroxybenzoic acid	C_7_H_6_O_3_	138.0317	leaves	GC-MS	[67]
**250**	Hydroxybenzoic acid	C_7_H_6_O_3_	138.0317	leaves	GC-MS	[67]
**251**	Dimethylbenzoic acid	C_9_H_10_O_2_	150.0681	leaves	GC-MS	[67]

IR: Infrared spectroscopy; ^13^C-NMR: Carbon-13 nuclear magnetic resonance spectrometry; ^1^H-NMR: Hydrogen-1 nuclear magnetic resonance spectrometry; MS: Mass spectrometry; HPLC-MS: High-performance liquid chromatography-mass spectrometry; UPLC-MS: Ultra performance liquid chromatography-mass spectrometry; GC-MS: Gas chromatography-mass spectrometry.

**Table 7:** Others isolated from *Eleutherococcus sessiliflorus.*

NO.	Name	Formula	Exact Theoretical Molecular Weight	Source	Characterization Method	Refs.
**272**	Frangulin B	C_20_H_18_O_9_	402.0951	leaves	UPLC-MS	[20]
**273**	Purpurin	C_14_H_8_O_5_	256.0372	leaves	UPLC-MS	[20]
**274**	Tyrosol	C_8_H_10_O_2_	138.0681	fruits	^13^C-NMR, ^1^H-NMR, MS	[48]
**275**	Protocatechuic aldehyde	C_7_H_6_O_3_	138.0317	fruits	^13^C-NMR, ^1^H-NMR, MS	[48]
**276**	Pyrocatechol	C_6_H_6_O_2_	110.0368	fruits	^13^C-NMR, ^1^H-NMR, MS	[48]
**277**	Phenol	C_6_H_6_O	94.0419	leaves	^13^C-NMR, ^1^H-NMR, MS	[48]
**278**	Salidroside	C_14_H_20_O_7_	300.1209	fruits	MS, ^13^C-NMR, ^1^H-NMR	[14]
**279**	4-Hydroxybenzyl-*β*-D-glucopyranoside	C_13_H_18_O_7_	286.1053	fruits	MS, ^13^C-NMR, ^1^H-NMR	[14]
**280**	2-Hydroxy-5-(2-hydroxyethyl) phenyl-O-*β*-D-glucopyranoside	C_14_H_20_O_8_	316.1158	fruits	MS, ^13^C-NMR, ^1^H-NMR	[14]
**281**	*β*-Sitosterol	C_29_H_50_O	414.3862	stems, fruits	IR, ^13^C-NMR, ^1^H-NMR, GC-MS	[54,69]
**282**	Daucosterol	C_35_H_60_O_6_	576.4390	stems, fruits, root barks, leaves	UPLC-MS, IR, ^13^C-NMR, ^1^H-NMR	[20,53,54]
**283**	Stigmasterol-3-O-*β*-D-glucopyranoside	C_35_H_58_O_6_	574.4233	stems	IR, ^13^C-NMR, ^1^H-NMR	[54]
**284**	Stigmasterol	C_29_H_48_O	412.3705	fruits	IR, ^13^C-NMR, ^1^H-NMR, GC-MS	[69]
**285**	Stigmast-5-en-3*β*,7*β*-diol	C_29_H_50_O_2_	430.3811	fruits	IR, ^13^C-NMR, ^1^H-NMR, GC-MS	[69]
**286**	19-Nortestosterone	C_18_H_26_O_2_	274.1933	leaves	UPLC-MS	[20]
**287**	Falcarindiol	C_17_H_24_O_2_	260.1776	root barks	^13^C-NMR, ^1^H-NMR	[53]
**288**	Cuminyl alcohol	C_10_H_14_O	150.1045	leaves	^13^C-NMR, ^1^H-NMR, MS	[48]
**289**	5-Hydroxymethylfurfural	C_6_H_6_O_3_	126.0317	fruits	IR, ^13^C-NMR, ^1^H-NMR	[22]
**290**	Raffinose	C_18_H_32_O_16_	504.1690	leaves	UPLC-MS	[20]
**291**	Sucrose	C_12_H_22_O_11_	342.1162	leaves	UPLC-MS, GC-MS	[20,67]
**292**	Maltol	C_6_H_6_O_3_	126.0317	leaves	UPLC-MS	[20]
**293**	*β*-Gentiobiose	C_12_H_22_O_11_	342.1162	leaves	GC-MS	[67]
**294**	D-Cellobiose	C_12_H_22_O_11_	342.1162	leaves	GC-MS	[67]
**295**	D-Fructose	C_6_H_12_O_6_	180.0634	leaves	GC-MS	[67]
**296**	D-Galactose	C_6_H_12_O_6_	180.0634	leaves	GC-MS	[67]
**297**	Galactose oxime	C_6_H_13_NO_6_	195.0743	leaves	GC-MS	[67]
**298**	D-Mannose	C_6_H_12_O_6_	180.0634	leaves	GC-MS	[67]
**299**	D-Gluconic acid	C_6_H_12_O_7_	196.0583	leaves	GC-MS	[67]
**300**	Levoglucosan	C_6_H_10_O_5_	162.0528	leaves	GC-MS	[67]
**301**	Arabinofuranose	C_5_H_10_O_5_	150.0528	leaves	GC-MS	[67]
**302**	L-Sorbopyranose	C_6_H_12_O_6_	180.0634	leaves	GC-MS	[67]
**303**	Methyl-*α-*D-ribofuranoside	C_6_H_12_O_5_	164.0685	leaves	GC-MS	[67]
**304**	D-Glucosamine	C_6_H_13_NO_5_	179.0794	fruits	IR, HPLC	[78]
**305**	Rhamnose	C_6_H_12_O_5_	164.0685	fruits	IR, HPLC	[78]
**306**	D-Glucuronic acid	C_6_H_10_O_7_	194.0427	fruits	IR, HPLC	[78]
**307**	D-Galacturonic acid	C_6_H_10_O_7_	194.0427	fruits	IR, HPLC	[78]
**308**	Glucose	C_6_H_12_O_6_	180.0634	fruits	IR, HPLC	[78]
**309**	(+)-Xylose	C_5_H_10_O_5_	150.0528	fruits	IR, HPLC	[78]
**310**	Fucose	C_6_H_12_O_5_	164.0685	fruits	IR, HPLC	[78]
**311**	1,2-Bis(trimethylsiloxy)cyclohexene	C_12_H_26_O_2_Si_2_	258.1471	leaves	GC-MS	[67]
**312**	Diphenyl(trimethylsilyl)phosphine	C_15_H_19_PSi	258.0994	leaves	GC-MS	[67]
**313**	Pentasiloxane	H_12_O_4_Si_5_	215.9582	leaves	GC-MS	[67]
**314**	Trisiloxane	H_8_O_2_Si_3_	123.9832	leaves	GC-MS	[67]

UPLC-MS: Ultra performance liquid chromatography-mass spectrometry; ^13^C-NMR: Carbon-13 nuclear magnetic resonance spectrometry; ^1^H-NMR: Hydrogen-1 nuclear magnetic resonance spectrometry; MS: Mass spectrometry; IR: Infrared spectroscopy; GC-MS: Gas chromatography-mass spectrometry; HPLC: High-performance liquid chromatography.

## Data Availability

All data presented in this study are available in the article.
